# The cacao gene atlas: a transcriptome developmental atlas reveals highly tissue-specific and dynamically-regulated gene networks in *Theobroma cacao* L

**DOI:** 10.1186/s12870-024-05171-9

**Published:** 2024-06-26

**Authors:** Evelyn Kulesza, Patrick Thomas, Sarah F. Prewitt, Akiva Shalit-Kaneh, Eric Wafula, Benjamin Knollenberg, Noah Winters, Eddi Esteban, Asher Pasha, Nicholas Provart, Craig Praul, Lena Landherr, Claude dePamphilis, Siela N. Maximova, Mark J. Guiltinan

**Affiliations:** 1 Department of Plant Science, The Pennsylvania State University, University Park, PA 16802 USA; 2 Department of Cell & Systems Biology, Centre for the Analysis of Genome Evolution and Function, University of Toronto, Toronto, ON Canada; 3 Huck Institute of the Life Sciences, The Pennsylvania State University, University Park, PA 16802 USA; 4 USDA Animal and Plant Health Inspection Service (APHIS), Riverdale, MD 20737 USA; 5 Plant Sciences, Volcani-ARO (Agricultural and Rural Organization), Gilat, Israel; 6 Children’s Hospital of Philadelphia, Philadelphia, PA 19104 USA; 7 Mars Inc, Davis, CA 95616 USA; 8 Battelle Memorial Institute, Columbus, OH 43201 USA

**Keywords:** Transcriptome atlas, Tissue-specificity, Cacao genomics, Gene expression

## Abstract

**Background:**

*Theobroma cacao*, the cocoa tree, is a tropical crop grown for its highly valuable cocoa solids and fat which are the basis of a 200-billion-dollar annual chocolate industry. However, the long generation time and difficulties associated with breeding a tropical tree crop have limited the progress of breeders to develop high-yielding disease-resistant varieties. Development of marker-assisted breeding methods for cacao requires discovery of genomic regions and specific alleles of genes encoding important traits of interest. To accelerate gene discovery, we developed a gene atlas composed of a large dataset of replicated transcriptomes with the long-term goal of progressing breeding towards developing high-yielding elite varieties of cacao.

**Results:**

We describe the creation of the Cacao Transcriptome Atlas, its global characterization and define sets of genes co-regulated in highly organ- and temporally-specific manners. RNAs were extracted and transcriptomes sequenced from 123 different tissues and stages of development representing major organs and developmental stages of the cacao lifecycle. In addition, several experimental treatments and time courses were performed to measure gene expression in tissues responding to biotic and abiotic stressors. Samples were collected in replicates (3–5) to enable statistical analysis of gene expression levels for a total of 390 transcriptomes. To promote wide use of these data, all raw sequencing data, expression read mapping matrices, scripts, and other information used to create the resource are freely available online. We verified our atlas by analyzing the expression of genes with known functions and expression patterns in Arabidopsis (*ACT7, LEA19, AGL16, TIP13, LHY, MYB2*) and found their expression profiles to be generally similar between both species. We also successfully identified tissue-specific genes at two thresholds in many tissue types represented and a set of genes highly conserved across all tissues.

**Conclusion:**

The Cacao Gene Atlas consists of a gene expression browser with graphical user interface and open access to raw sequencing data files as well as the unnormalized and CPM normalized read count data mapped to several cacao genomes. The gene atlas is a publicly available resource to allow rapid mining of cacao gene expression profiles. We hope this resource will be used to help accelerate the discovery of important genes for key cacao traits such as disease resistance and contribute to the breeding of elite varieties to help farmers increase yields.

**Supplementary Information:**

The online version contains supplementary material available at 10.1186/s12870-024-05171-9.

## Background

*Theobroma cacao*, the chocolate tree, is an evergreen, neotropical, understory tree (*Malvaceae*), closely related to other economically important crops such as cotton, hibiscus, okra, and durian. *T. cacao* is native to the Amazon and is also cultivated in tropical latitudes of Central and South America, the Caribbean, Africa, and Asia as a cash crop [1]. Its widespread cultivation yielded over 5.240 million tons and generated $10 billion dollars in the global commodity market in 2021, making it an economically important crop [2]. The transformation of cocoa seeds into chocolate supports a $170 billion dollar per year global industry [2]. Cacao is often grown by smallholder farmers, and improving cacao cultivation can bring social benefits to farmers by providing income diversification and, in some cases, an alternative to illicit crop cultivation in countries such as Colombia and Peru. Diversified agroforestry systems are among the best cacao farming practice; providing shade and other benefits to cacao and ecosystems such as soil and water stabilization, and habitat provision for migratory birds, insects, amphibians, and other species [3–8].

**Fig. 1:**
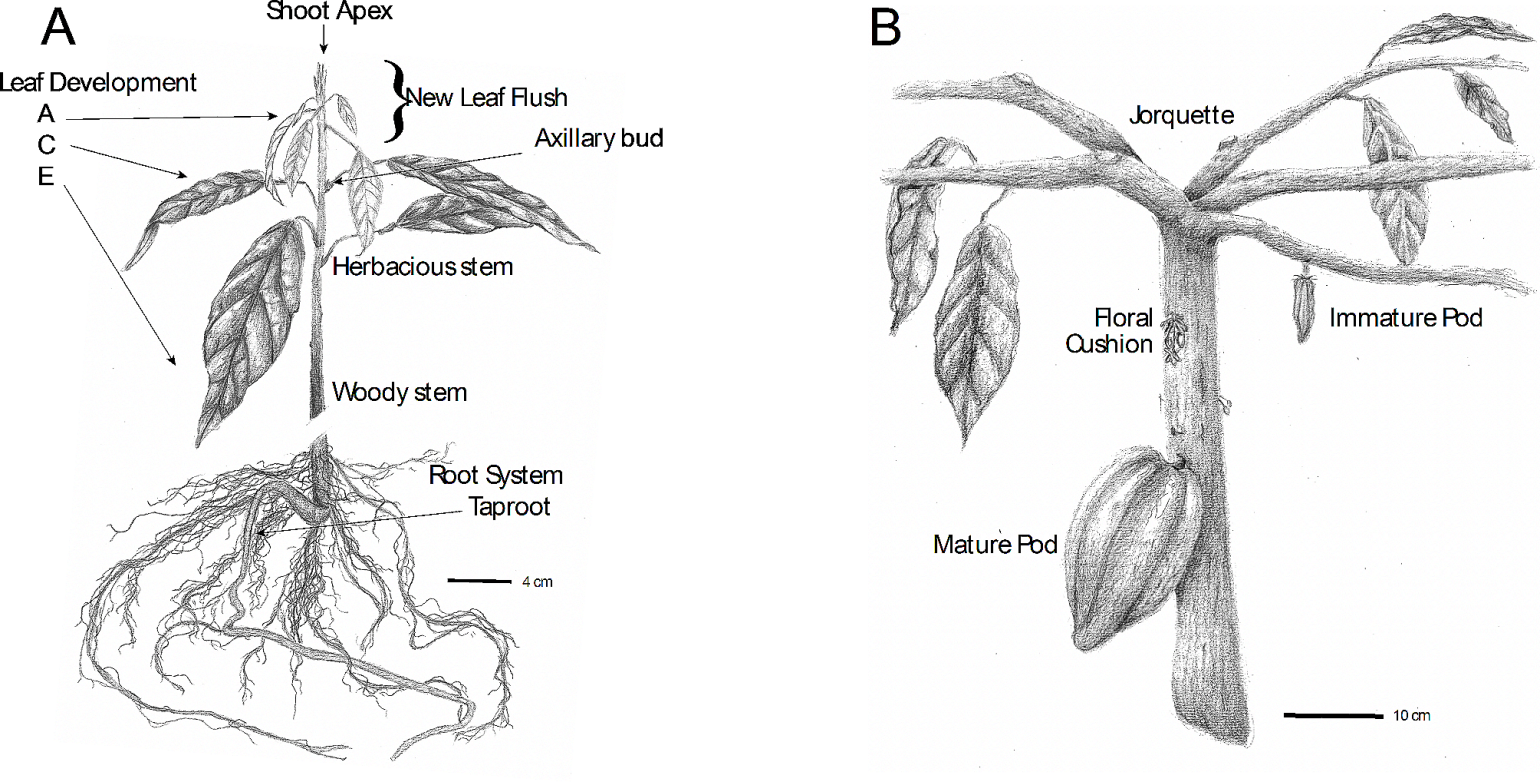
Botanical illustration of orthotropic and plagiotropic cacao plants. Illustrations showing (**A**) a 6-month-old orthotropic plant undergoing a new leaf flush and (**B**) the trunk and jorquette of a mature plagiotropic plant growing fruits and flowers. Orthotropic plants grow vertically, and produce leaves with spiral phyllotaxy, while plagiotropic plants form five branches growing at a fixed angle from vertical growth and producing leaves with alternate phyllotaxy (most leaves in image removed to highlight jorquette structure). Clusters of inflorescences form on main trunks or branches (commonly called floral cushions). A mature fruit (fruit) is depicted, a cross section and sub-tissues are shown in Fig. 2A. Scale bars are indicated in cm

Cacao is a valuable commodity crop with unique characteristics. The growth of a cacao seedling can be divided into two distinct stages: orthotropic and plagiotropic growth. During the juvenile orthotropic growth stage (Fig. 1A), seedlings produce stems and leaves in a vertical manner, characterized by a single dominant apical meristem and leaves with a spiral arrangement [9–11]. Plagiotropic growth (Fig. 1B) is the second vegetative state and occurs after a phase change, where the apical meristem is lost, and the seedling produces five branches from the subtending axillary meristems. These branches have active vegetative apical meristems that produce leaves with an alternate arrangement growing upwards at an angle of approximately 25–65 degrees from the horizontal, forming a branching architecture commonly referred to as jorquette. Soon afterwards, some of the axillary meristems further down on the main stem transition to reproductive development, the plant starts to produce flowers and fruits on its main stem and near the bases of the jorquette branches.

Leaves grow on both orthotropic and plagiotropic shoots with alternating phases of active and dormant vegetative meristem development lasting about 60 days [12]. During the flushes, meristems produce leaf primordia rapidly for about 20 days, producing about 11 leaves which proceed through developmental stages A-E, after which the meristem enters a dormancy period lasting another 20 days during which the initial leaf primordia and young leaves (Stages A-D) develop to maturation (Stage E). After about 16 days of dormancy, the meristems will begin to produce leaf primordia again. In the field, under well-watered and normal temperature conditions, this cycle will continue to repeat in all growing shoot tips, indefinitely. However, the initiation of flushing is repressed under drought and cold conditions, while rain after drought conditions and warmer conditions activate flushing. Succeeding the transition from orthotropic to plagiotropic vegetative growth, multiple dormant axillary meristems on the main vertical trunks (and as the tree ages, on major branches) are activated and transition developmentally from vegetative to floral meristems, likely under the control of the development regulating co-transcription factor *FT* (florigen) [13] (Fig. 1B). Flowering completes the phase change and enables the tree to produce fruit for up to a century or longer.

*T. cacao* seeds (cocoa beans) are ground and used to produce cocoa solids and cocoa butter. Both cocoa solids and butter are used in making chocolate and confectionaries, while cocoa butter has applications in the cosmetic and pharmaceutical industries for use in skincare products and oral tablets, respectively. Cacao’s lipid composition, which causes it to be solid at room temperature and melt at human body temperature, is highly prized [14]. As global demand for cacao continues to grow along with the world population, it is becoming increasingly important to develop sustainable farming practices that are both economically viable and environmentally sound while moving away from current production methods which rely on unsustainable and climate-unfriendly deforestation. One approach to making cacao cultivation a more economically viable and environmentally sustainable practice is to develop high-efficiency, sustainable cacao farming systems which implement best agronomic practices and genetically improved cacao varieties. While there is some progress on this front, breeding a new cacao variety to farm release requires about 20–30 years. One means of accelerating the development of elite cacao varieties is to create a gene expression atlas for accelerating gene discovery, developing molecular-assisted selection techniques, and facilitating genome editing and speed-breeding systems.

Although genetic research on cacao has been slow compared to major crops, a considerable proliferation of cacao genomic resources has enabled breeders and researchers to consider cacao to be a model tropical tree crop. The genome of *T. cacao* was first sequenced and assembled in 2010 from the Criollo genotype [15], followed by the Matina 1–6 cultivar in 2013 [16]. In 2017, the Criollo genome assembly was updated and improved using exclusively next generation sequencing (NGS) technologies to fill in gaps and correct mis-assemblies [17]. Single nucleotide polymorphism (SNP) data has also been gathered and used for 200 cacao genotypes, providing representation for ten genetic groups and a resource for population genetics research [18]. Most recently, 31 genome assemblies from wild cacao accessions across the four genetic groups were published, further increasing the wealth of cacao genomic resources [19, 20]. An additional set of seven high quality *de novo* assembled genomes were recently developed and will be released to the public as well [19, 20]. These advances have enabled cacao science to keep pace in a scientific world where “big data” and “-omics” analyses of plant biology are being developed at a staggering pace.

All life depends on coordinated expression of large gene sets following a developmental program; said genes are also responsive to myriad biotic and abiotic interactions. Understanding gene regulation has been a central focus of molecular genetics research over the past 50 years. Recent advancements in high-throughput DNA sequencing and biocomputing have enabled a new era of large dataset-driven science. Transcriptomic data is an invaluable resource for understanding gene expression in an organism at specific points in time and space. A transcriptome dataset provides estimated relative RNA levels for all expressed genes in sampled tissues and can provide unambiguous evidence needed to determine gene structures and exploration of gene regulation during development and in response to external stimuli [21–23]. A gene atlas is a database resource that holds millions of datapoints, representing a large matrix of gene expression levels, which can be explored using various biocomputing and statistical approaches to discover key genes involved in different pathways, explore expression of a gene across different tissues, and study gene regulation without the need to perform time-intensive and expensive gene expression experiments. Although transcriptomic resources for cacao have been developed, they have been limited in scope and largely without replications, severely limiting their utility for gene discovery. Access to the cacao transcriptome atlas will provide cacao scientists with resources to accelerate molecular biology and breeding to improve cacao, while scientists in the broader genomics community can use it for comparative studies of trees and tropical crops.

Here, we report a comprehensive transcriptome study of RNA sequences from *T. cacao* including (1) a developmental time course covering multiple life stages, including vegetative and reproductive tissues, (2) a drought and diurnal time course of vegetative tissues, and (3) a leaf infection atlas examining cacao’s response to *Phytophthora megakarya* infection in both resistant and susceptible genotypes. We generated transcriptome read mapping data and tissue images, which were then uploaded to the Bio-Analytic Resource for Plant Biology (BAR) database, resulting in a graphic expression atlas that can be easily accessed by inputting a query gene [24]. This study’s objective was to provide a wide-ranging resource of cacao transcriptomic data that could be used for preliminary discovery of any expressed gene in cacao. To validate our dataset, we analyzed the expression of cacao orthologs of well-studied Arabidopsis genes with distinct expression profiles. We also performed global transcriptome analysis, with a particular focus on tissue-specific gene expression across different developmental stages and organ types. Through this analysis, we identified numerous genes that exhibit profound tissue- and organ-specificity, expressed in cacao at levels ranging from low to very high.

## Results

### The Cacao eFP Browser

Pictographic representations of a gene’s expression level could help generate hypotheses about gene function, accelerate data mining, and provide user-friendly access. We have developed three different cacao electronic Fluorescent Pictograph (eFP) Browsers based on eFP Browser framework described by Winter et al. [25]. For these eFPs, data was mapped to three different genotypes, Scavina 6 (SCA6), CCN-51 and to a Criollo genotype (B97-61/B2) abbreviated as TC. The Cacao SCA eFP Browser (https://bar.utoronto.ca/efp_cacao_sca/cgi-bin/efpWeb.cgi) contains 4 views: a seed atlas, a meristem atlas, a developmental atlas, and a drought and diurnal atlas. The Cacao CCN-51 eFP Browser (https://bar.utoronto.ca/efp_cacao_ccn/cgi-bin/efpWeb.cgi) encompasses two views, a developmental atlas, and a drought and diurnal atlas. The Cacao TC eFP Browser (https://bar.utoronto.ca/efp_cacao_tc/cgi-bin/efpWeb.cgi) comprises two views, an Infected C stage Leaf Atlas (for Scavina6 and NA32 varieties [26], C stage leaves infected with *Phytophtora megakarya* for several timepoints) up to 72 hours (h) and a *T. cacao* Leaf Development Atlas (for Scavina6 and ICS-1 varieties, A-E stages leaves). Counts per million (CPM) were used for the CCN51 and SCA6 browsers, in the TC browser the leaf development atlas uses CPM counts whereas the leaf infection atlas uses Transcript per million (TPM) counts. Genes may be queried one at a time using the absolute or relative expression modes, which show respectively the absolute expression level of that gene in the tissues or the expression level relative to a control value. To look at the relative expression of two genes, the compare expression mode shows the log2fold change in expression between the two selected genes. In addition to the browser view, there is also a table and bar chart of expression values from the mean of the replicates to further quantify expression values. Annotations of the gene IDs can be found at [20].

### Analysis of cacao gene expression in response to biotic and abiotic interactions

The Cacao Atlas includes data from several experimental treatments, including two experiments where plants were grown with and without pathogen inoculation. These experiments reported elsewhere [20, 26], involve multiple genotypes with known disease resistance classes. In both experiments, plants were inoculated on leaves, and RNA was extracted after infection, allowing for the identification of candidate disease resistance genes and mechanisms of resistance. The disease resistance data is described further in [27].

Additionally, the Atlas contains a new dataset from a time course experiment in which seedlings were treated with or without drought stress, and samples were taken from three different tissues (apices, leaves, and roots) every 4 h over 24 h. This data provides an opportunity to investigate the transcriptional response of cacao tissues to drought and how gene expression changes over the course of a day, as well as to explore the interaction between these factors.

### Analysis of gene expression during the life cycle of *Theobroma cacao*

To validate our atlases, we assessed the expression of six genes with well-known expression profiles in Arabidopsis based on reciprocal best hits from the cacao SCA6 genes. The genes included: *Actin* (*ACT7*; SCA-6_Chr1v1_01159), *Late Embryogenesis Abundant protein 19* (*LEA19*; SCA-6_Chr10v1_26676), *Agamous-like 16* (*AGL16*; SCA-6_Chr3v1_08706), *Gamma-tonoplast intrinsic protein 3* (*TIP13*; SCA-6_Chr9v1_26190), *Late Elongated Hypocotyl* (*LHY*; SCA-6_Chr1v1_03181), and a *MYB domain protein 2* (*MYB2*; SCA-6_Chr4v1_13194). We chose *ACT7* because it is constitutively expressed in all eukaryotes [28]. The other genes were selected based on their unique tissue- or condition-specific expression patterns discussed below (Table 1) [29–33]. Cacao orthologs were identified using a BLASTp search in a Blast2GO pipeline described in Materials and Methods. In addition, principal component analyses (PCAs) were performed to identify any outliers in the sampled replicates. Clustering of samples indicates no major outliers (Additional File 1).

**Table 1:** Genes used for the *T. cacao* atlas validation

Gene Name	Gene Abbreviation	*T. cacao* Gene ID	Arabidopsis Gene ID	Gene Function
*Actin*	*ACT7*	SCA-6_Chr10v1_26940	AT5G09810	Production of actin for use in the cytoskeleton
*Late embryogenesis abundant protein 19*	*LEA19*	SCA-6_Chr10v1_26676	AT2G40170	Response to abiotic stress, tolerance to dehydration
*Agamous-Like 16*	*AGL16*	SCA-6_Chr3v1_08706	AT3G57230	*MADS*-box transcription factor regulating flowering time
*Gamma-tonoplast intrinsic protein 3*	*TIP13*	SCA-6_Chr9v1_26190	AT4G01470	Aquaporins that facilitate transmembrane travel of water and small uncharged molecules
*MYB domain protein 2*	*MYB2*	SCA-6_Chr4v1_13194	AT4G13480	Transcription factor that regulates salt and drought stress response

**Fig. 2:**
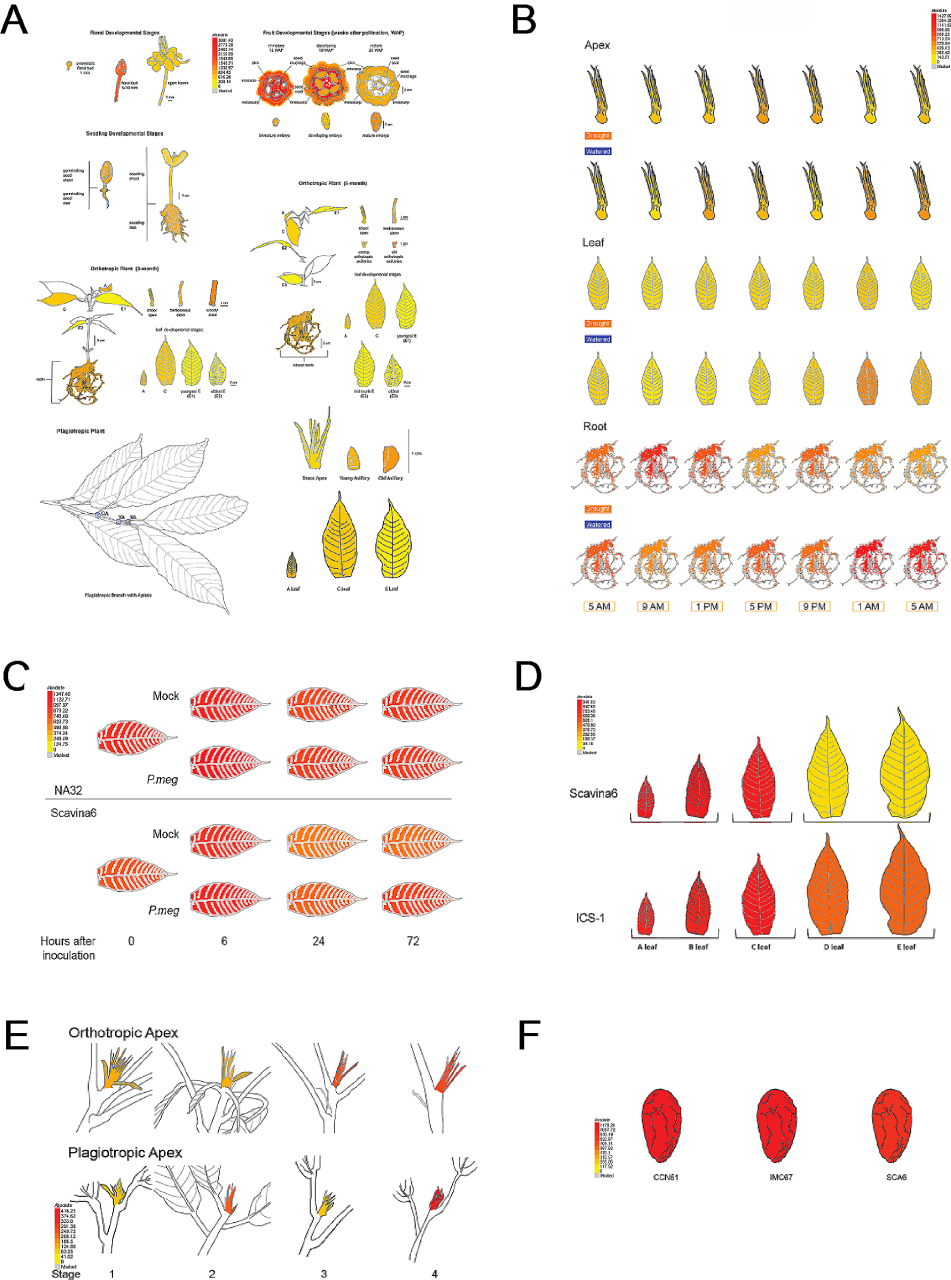
Gene expression profile of cacao actin gene. Sub-atlases represented in the cacao gene atlas. Tissues represented across the six sub-atlases that compose the Cacao Gene Atlas including (**A**) the developmental atlas, (**B**) drought-diurnal atlas, (**C**) leaf infection atlas, (**D**) leaf development atlas, (**E**) meristem atlas, and (**F**) seed atlas. The expression of *Actin* (*ACT7*) is depicted, generally considered constitutively expressed in all tissues. Tissues are colored according to the mean number of mapped reads per million (CPM) of replicate samples. Color scale is depicted in each sub-atlas

**Fig. 3:**
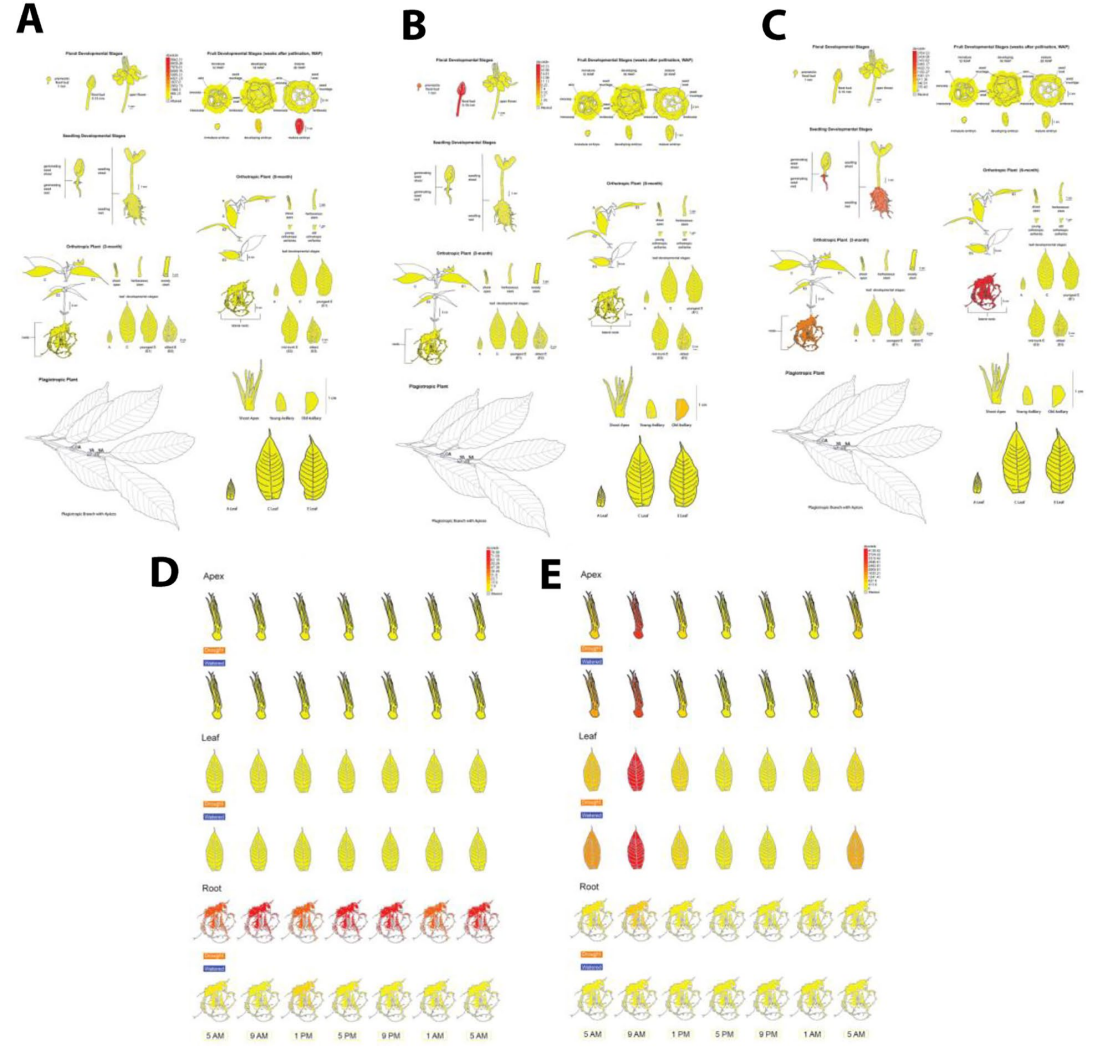
Atlas validation gene expression via eFP browser. Gene expression as view in the BAR eFP browser of (**A**) *Late embryogenesis abundant 19* (*LEA19*), expressed in late seed development and in response to drought, (**B**) *Agamous-like 16* (*AGL16*), a transcription factor involved in specification of floral organ identity, (**C**) *Gamma-tonoplast intrinsic protein 3* (*TIP13*), a root specific aquaporin transporter, (**D**) *Late elongated hypocotyl* (*LHY*), which is a transcription factor involved in regulating circadian rhythm and (**E**) a *MYB* transcription factor (*MYB2*), which regulates dehydration response in plants. A-C are represented by the Developmental Atlas, while D-E are the Drought and Diurnal Atlas. Tissues are colored according to the mean number of mapped reads per million (CPM) of replicate samples. Color scale is depicted in each sub-atlas

**Fig. 4:**
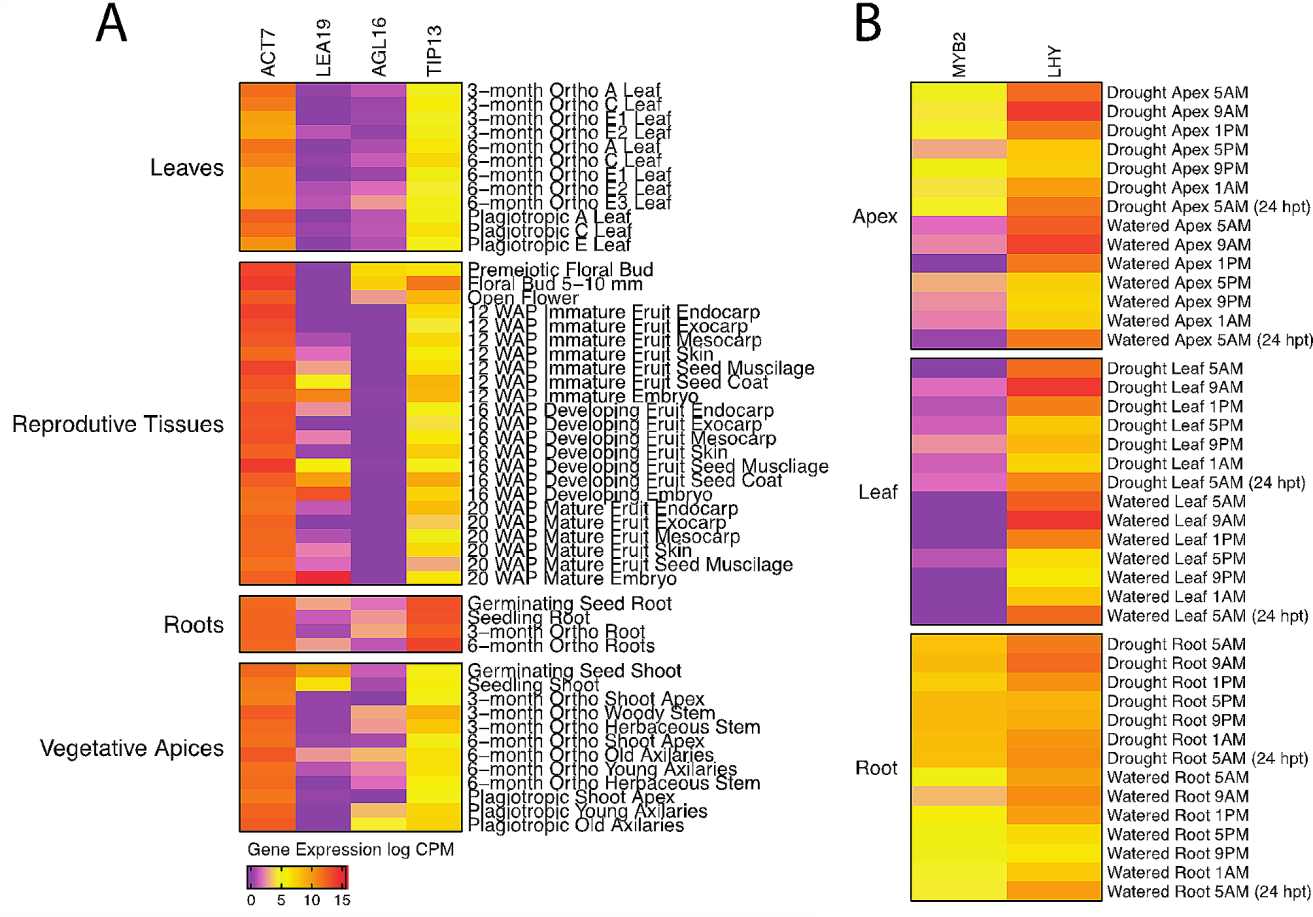
Validation of gene expression patterns with genes of known and highly conserved expression profiles. **(A)** Log2 of mean number of mapped reads per million for the developmental atlas. Genes represented include *Actin* (*ACT7*), generally considered constitutively expressed in all tissues, *Late embryogenesis abundant 19* (*LEA19*), expressed in late seed development and in response to drought, *Agamous-like 16* (*AGL16*), a transcription factor involved in specification of floral organ identity, and *Gamma-tonoplast intrinsic protein 3* (*TIP13*), a root specific aquaporin transporter **(B)** Log2 of mean number of mapped reads per million read for the drought/diurnal atlas. Genes represented include a *MYB* transcription factor (*MYB2*), which regulates dehydration response in plants, and *Late elongated hypocotyl* (*LHY*), which is a transcription factor involved in regulating circadian rhythm

Among the six genes we analyzed in our validation study, we found four had similar expression patterns in Arabidopsis and cacao while two had divergent functions (Figs. 2A - F, 3A-F and 4A-F). In our developmental atlas, *ACTIN7* (SCA-6_Chr1v1_01159), known for constitutive expression across tissues in higher plants [28] was expressed in all sampled tissue types (Fig. 2A-F). Expression values ranged from 25 to 442 CPM, with expression values ranging from 100 to 300 CPM in most tissues; these values show expression across all cacao tissues and exhibit a similar expression pattern found in other higher plants. *LATE EMBRYOGENESIS PROTEIN 19* (*LEA19;* SCA-6_Chr10v1_26676) had low or no expression across most tissues but was abundant (9842 CPM) in cacao mature embryos (Fig. 3A). This expression pattern is consistent with Arabidopsis where *LEA* is expressed only and abundantly during late seed development [30, 34].

We also evaluated the gene expression of two genes with known roles in drought-diurnal response: *LATE ELONGATED HYPOCOTYL* (*LHY*) and *MYB DOMAIN PROTEIN 2* (*MYB2)*. *LHY* is a circadian clock gene known to be expressed in Arabidopsis leaves and stems before and shortly after dawn [35–37]. In the drought and diurnal atlas, we observed that *TcLHY* (SCA-6_Chr1v1_03181) expression start to rise at around 5 AM and appears to peak at the 9 AM timepoint for leaf, apical, and root tissues. We also observed the highest level of expression in apex and leaf tissues; leaves had the highest expression level at 4138.02 CPM, followed by apical tissues at 3099.71 CPM with roots having the lowest level of expression at 786.04 CPM (Figs. 3E and 4). Under long day conditions in Arabidopsis leaves, *LHY* expression is induced upon the middle of the dark cycle and peaks at the start of the light cycle after 12 h of plants being in the dark (0–5277 read counts across all time points) [38]. *TcMYB2* (SCA-6_Chr4v1_13194) was observed to be mainly expressed in drought-stressed roots (200–400 CPM across the time points) with some low-level expression in drought-stressed apices and leaves (Fig. 3D Fig. 4). We also observed that its peak expression was earlier in the time course (9 AM) in both the apex and leaf tissues. In Arabidopsis and other species, *MYB2* is known to be expressed in leaves under drought and in roots under salt stress [39]. Under drought conditions in Arabidopsis leaves, *MYB2* was at its peak expression later in the time course (4 days after onset of drought conditions; late day) (18–45 read counts) [40]. While we found *LHY* diurnal expression to be similar in both cacao and Arabidopsis leaves, we found differences in the magnitude and temporal expression of cacao and Arabidopsis *MYB* under drought conditions with cacao *MYB2* being expressed earlier and at a greater magnitude.

**Fig. 5:**
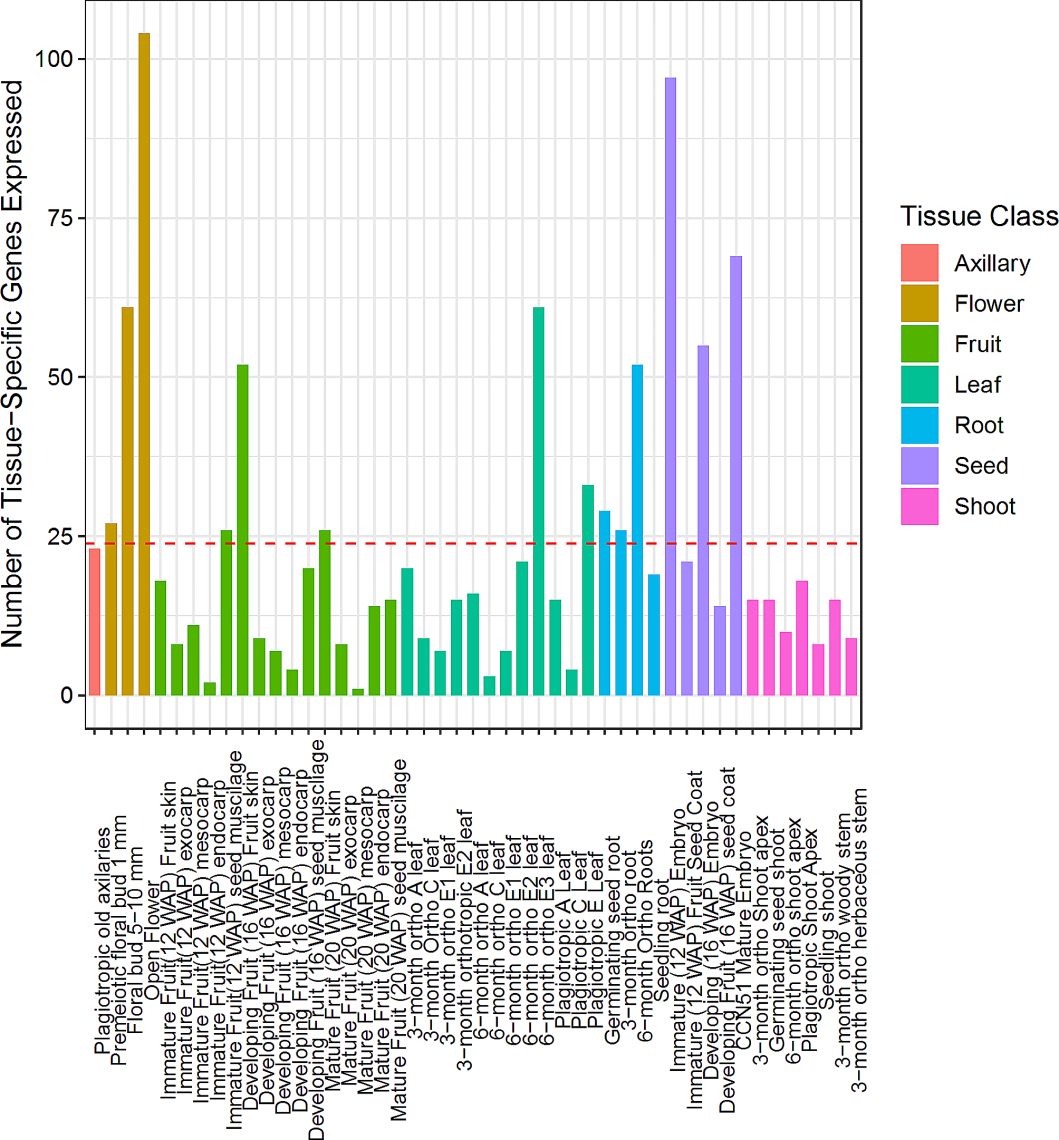
“Extremely” Tissue-specific genes in the *T. cacao* gene atlas. Bar plot displaying the number of “extremely” tissue-specific genes expressed above 30 CPM for each tissue type in the *T. cacao* gene atlas. Genes were identified as expressed if they reached a read count threshold greater than 30 CPM in most replicates as described in the Methods. Genes were defined as “extremely” tissue-specific if they met the 30 CPM threshold and were not identified as expressed in another tissue type at that threshold. Red dashed line represents the mean number of tissue-specific genes per library in the *T. cacao* gene atlas

Two of the genes we examined for atlas validation did not exhibit expression profiles similar to their best hit in Arabidopsis. *AGAMOUS-LIKE 16* (*AGL16*), is known to be expressed in the roots, stems, and mature leaves in Arabidopsis [31]. *TcAGL16* (SCA-6_Chr3v1_08706) had low or no expression in most tissues (Fig. 3B–5 CPM). The highest expression was observed in the pre-meiotic and 5–10 mm floral buds (Fig. 3B) with 133.25 and 186.75 CPM (Fig. 4). The expression profiles of these two genes were not similar. *GAMMA-TONOPLAST INTRINSIC PROTEIN 3* (*TIP13*) which has roles in water transport in Arabidopsis is known to be expressed in floral tissues and pollen [37]. In cacao, we observed *TIP13* (SCA-6_Chr9v1_26190) expression in several root and floral tissues. Expression was high in root tissues and low in other developmental atlas tissues, such as fruit tissue, floral tissues, and stem tissues (Figs. 3C and 4). These expression patterns were quite distinct compared to those observed in Arabidopsis for *TIP13*, suggesting some differences in their functions. In Arabidopsis, *TIP13* was expressed at very low levels in.

all tissues except in flowers in leaves under drought conditions. While the low levels of expression in most floral tissues is consistent with Arabidopsis findings, cacao’s root, fruit, and stem expression patterns indicate the potential for neofunctionalization of *TcTIP13*.

### Numbers of expressed genes in different Cacao tissues

At the 1 CPM threshold, we identified an average of 15,273 expressed genes among the 51 tissue types in the atlas, which represents approximately 53.9% of all genes in the cacao genome (Table 2). Tissues with the highest number of expressed genes included root, apex, and axillary tissues, such as plagiotropic old axillaries (18,594 genes), 6-month orthotropic shoot apices (18,234 genes), 3-month orthotropic roots (17,338 genes), seedling roots (17,243 genes), and 3-month orthotropic shoot apices (17,128 genes). Tissues with the lowest number of expressed genes included immature fruit (12 WAP) skins (13,330 genes), premeiotic floral buds (1 mm) (12,894 genes), mature fruits (20 WAP) seed mucilage (12,225 genes), open flowers (9,214 genes), and floral buds (5–10 mm) (8,631 genes). We repeated the analysis using a 30 CPM threshold and found an average of 5,180 genes expressed in all tissue types, which accounts for 18.3% of genes in the cacao genome (Table 2). Using this threshold, tissues with the most expressed genes included plagiotropic old axillaries (7,013 genes), 6-month orthotropic shoot apices (6,803 genes), plagiotropic shoot apices (6,106 genes), 3-month orthotropic shoot apices (6,028 genes), and 3-month orthotropic A stage leaves (5,873 genes), while those with the fewest genes expressed included immature fruit (12 WAP) endocarp (4,038 genes), floral buds (5–10 mm) (3,978 genes), mature fruit (20 WAP) seed mucilage (3,877 genes), open flowers (3,865 genes), and immature fruit (12 WAP) seed mucilage (3,859 genes). We consider that genes expressed below 30 CPM in a single sample represent mostly background noise and conclude that gene expression levels below 30 CPM should be treated with caution as they may represent background noise resulting from very low levels of transcription.

**Table 2:** *Theobroma cacao* Gene Atlas read count table

Tissue Type	Genes > 1 CPM	Genes > 30 CPM	% Genes > 1 CPM	% Genes > 30 CPM
3-month ortho Shoot apex	17,128	6028	0.60550783	0.213101425
Germinating seed root	16,910	5140	0.59780111	0.181708912
Germinating seed shoot	16,201	4937	0.572736593	0.174532471
Floral bud 5–10 mm	8631	3978	0.305122494	0.140629971
3-month Ortho C leaf	16,261	5474	0.574857708	0.193516456
3-month ortho E1 leaf	15,269	4739	0.539788595	0.167532789
Open Flower	9214	3865	0.325732669	0.136635203
3-month orthotropic E2 leaf	14,932	4755	0.527874996	0.16809842
Premeiotic floral bud 1 mm	12,894	4966	0.455827765	0.175557677
3-month ortho root	17,338	5721	0.612931735	0.202248383
3-month ortho woody stem	16,508	5321	0.583589635	0.188107611
3-month ortho herbaceous stem	16,837	5463	0.595220419	0.193127585
CCN51 Mature Embryo	14,619	4721	0.516809842	0.166896454
Seedling root	17,243	5656	0.609573302	0.199950507
Seedling shoot	16,677	5185	0.589564111	0.183299749
3-month ortho A leaf	16,711	5873	0.590766076	0.207621876
Developing Fruit (16 WAP) seed coat	15,020	5348	0.530985965	0.189062113
Plagiotropic old axilaries	18,594	7013	0.657333758	0.247923074
6-month ortho old axilaries	16,120	5088	0.569873087	0.179870612
6-month ortho young axilaries	16,804	5484	0.594053806	0.193869976
6-month ortho shoot apex	18,234	6803	0.644607063	0.240499169
6-month ortho herbaceous stem	16,755	5693	0.592321561	0.201258529
6-month ortho A leaf	16,592	5779	0.586559197	0.204298794
6-month ortho C leaf	16,266	5552	0.575034468	0.196273907
6-month ortho E1 leaf	15,256	5174	0.53932902	0.182910878
6-month ortho E2 leaf	15,313	5386	0.54134408	0.190405487
6-month ortho E3 leaf	15,665	5716	0.553787959	0.202071623
Mature Fruit (20 WAP) endocarp	13,495	4760	0.477074274	0.168275179
Mature Fruit (20 WAP) exocarp	14,128	4987	0.499452045	0.176300067
Mature Fruit (20 WAP) mesocarp	14,336	5011	0.506805246	0.177148513
Mature Fruit (20 WAP) Fruit skin	14,977	5405	0.529465832	0.191077173
Mature Fruit (20 WAP) seed muscilage	12,225	3877	0.432177325	0.137059427
Developing Fruit (16 WAP) endocarp	14,478	5028	0.51182522	0.177749496
Developing Fruit (16 WAP) exocarp	14,573	5137	0.515183653	0.181602856
Developing Fruit (16 WAP) mesocarp	15,011	5138	0.530667798	0.181638208
Developing Fruit (16 WAP) Fruit skin	14,836	5273	0.52448121	0.186410719
Developing Fruit (16 WAP) seed muscliage	14,805	4887	0.523385301	0.172764874
Immature Fruit (12 WAP) endocarp	13,897	4038	0.49128575	0.142751087
Immature Fruit (12 WAP) exocarp	13,699	4121	0.484286068	0.145685297
Immature Fruit (12 WAP) mesocarp	14,059	4371	0.497012762	0.154523279
Immature Fruit (12 WAP) Fruit skin	13,330	4049	0.471241206	0.143139958
Immature Fruit (12 WAP) seed muscilage	13,974	3859	0.494007848	0.136423092
Plagiotropic Shoot Apex	16,278	6106	0.575458691	0.215858875
Plagiotropic A Leaf	16,351	5590	0.578039382	0.19761728
Plagiotropic C Leaf	16,153	5473	0.5710397	0.193481104
Plagiotropic E Leaf	15,593	5276	0.55124262	0.186516774
6-month Ortho Roots	16,982	5501	0.600346449	0.194470958
Developing (16 WAP) Embryo	14,952	5392	0.528582034	0.190617598
Immature (12 WAP) Embryo	15,550	5774	0.549722487	0.204122035
Immature (12 WAP) Fruit Seed Coat	14,563	4572	0.514830134	0.161629017
Plagiotropic Young Axilaries	16,675	5677	0.589493407	0.200692898

### Analysis of spatial and temporally regulated Gene expression

The Cacao Gene Atlas is a powerful resource that enables us to explore genome-wide networks of genes that are co-regulated in response to tissue- or organ-specific patterns, as well as biotic or abiotic external stimuli. To identify networks of co-regulated genes, we used two levels of specificity. The first level is extremely-specific, where genes are expressed at > 30 CPM exclusively in one sample type and < 30 CPM in all other samples. This is a highly stringent definition. To identify genes that are functionally tissue-specific, we defined a second level of specificity. These genes have expression > 30 CPM in each tissue and greater than two-fold the mean expression of all other tissues combined [X _tissue_ > 2(X _all other tissues_)]. Although these genes may be expressed specifically in more than one tissue or stage of development of an individual tissue type, they are expressed at least 2-fold more than in a specific individual tissue or stage.

**Fig. 6:**
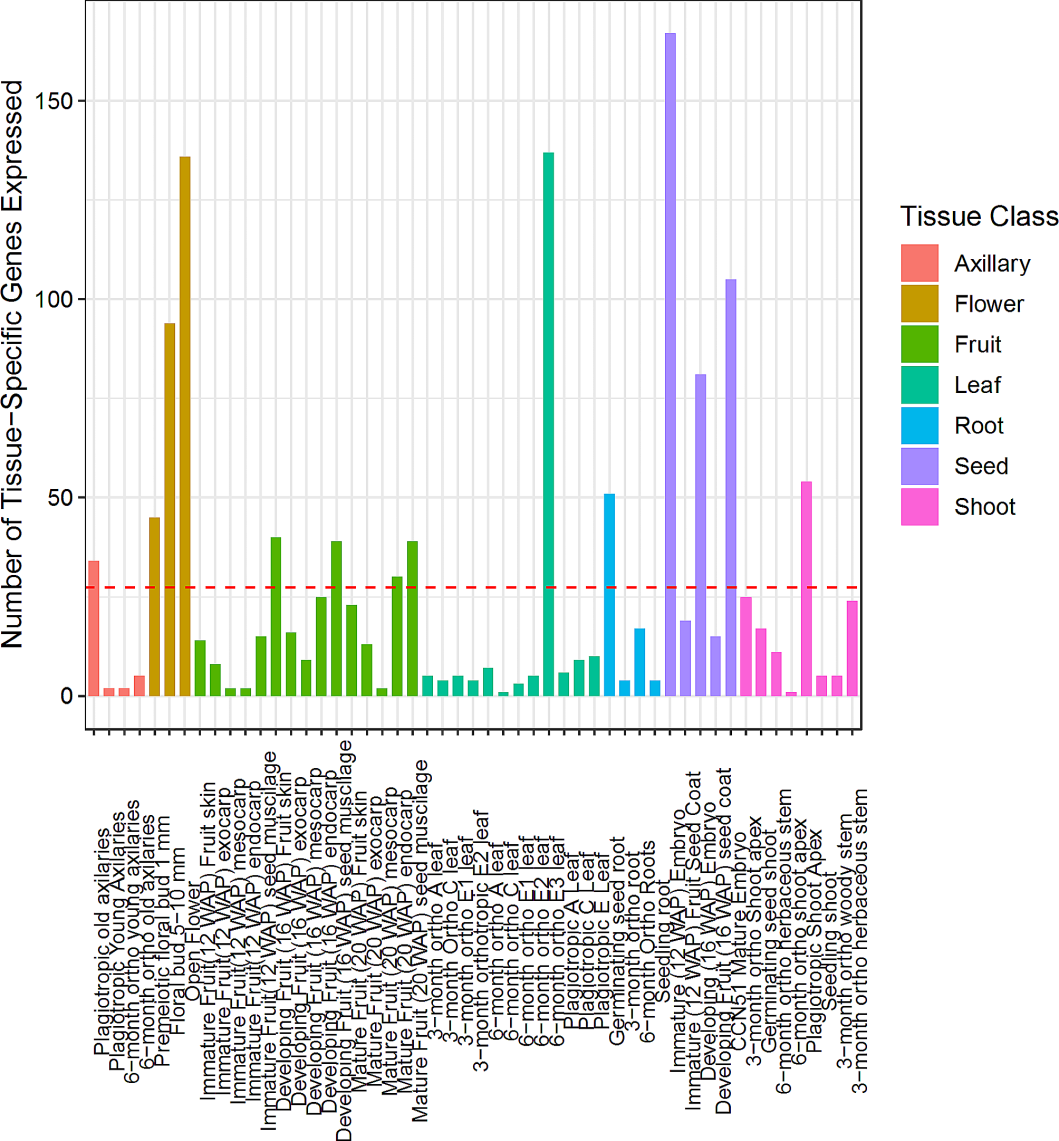
“Functionally” Tissue-specific genes in the *T. cacao* gene atlas. Bar plot displaying the number of “functionally” tissue-specific genes expressed above 30 CPM for each tissue type in the *T. cacao* gene atlas. Genes were identified as expressed if they reached a read count threshold greater than 30 CPM in most replicates as described in the Methods. Genes were defined as “functionally” tissue-specific if they met the 30 CPM threshold and their mean expression in said tissue was twice the global expression exclusion said tissue for the same gene. Red dashed line represents the mean number of tissue-specific genes per library in the *T. cacao* gene atlas

We detected 1119 genes that were extremely specific to a tissue type, with an average of 22 such genes per tissue type. These genes were identified in 47 out of 51 sample types (refer to Additional File 2A and 2C). Open flowers had the highest number of tissue-specific genes (104), followed by immature (12 WAP) embryos (97 genes), CCN-51 mature embryos (69 genes), large floral buds and 6-month-old orthotropic E3 leaves (61 genes) (Fig. 5). Notably, the number of tissue-specific genes increased as flower development progressed (premeiotic floral bud – 27 genes; floral bud – 61 genes, open flower – 104 genes). When we analyzed functionally tissue-specific genes, we identified 1396 tissue-specific genes in the *T. cacao* gene atlas: the immature (12 WAP) embryo had the highest number of functionally tissue-specific genes with 167 genes, followed by the 6-month-old orthotropic E3 leaf (137 genes), the open flower (136 genes), the CCN-51 mature embryo (105 genes), and the floral bud (94 genes) (refer to Fig. 6; Additional File 2B and 2C). Similar to the extremely tissue-specific genes, the number of tissue-specific genes also increased as development progressed in floral tissues (premeiotic floral bud – 45 genes; floral bud – 94 genes; open flower - 136 genes).

To investigate tissue-specificity more broadly, we combined data from samples of the same seven organ classes (axillary bud, flower, leaf, fruit, seed, root, or shoot) in the gene atlas (Additional File 3A) and identified “extremely organ-specific” and “functionally organ-specific” genes. We found that the number of organ-specific (OS) genes was increased against both extremely tissue-specific (from 1119 TS to 2328 OS genes) and functionally tissue-specific (from 1396 TS genes to 3905 OS genes) with extremely- and functionally-specific used as previously defined (Additional File 3B and Additional File 3C). In both cases, the leaf tissues had the highest number of OS genes (N _Extreme OS genes_ = 766; N _Functional OS genes_ = 960), and among all seven organ classes, we identified an average of 333 genes among the extremely OS genes and 558 functionally OS genes, with at least a two- or threefold difference in the number of specific genes identified by each method in a respective organ (Additional File 3D).

To determine if the organ-specific genes were enriched for functional classes of genes, we performed Gene Ontology (GO) enrichment analysis of the “functionally” organ-specific genes identified in seven organ classes and found enriched GO terms in all of them. Leaves had the largest number of enriched GO terms, with 44 terms identified across three GO categories (biological process, molecular function, and cellular component). Among the GO terms associated with biological processes, we found that the most overrepresented among “functionally” leaf-specific genes included " chloroplast thylakoid membrane”, “chloroplast stroma”, " chloroplast envelope”, " photosynthesis”, “plastoglobule” and “photosystem II” (Additional File 3E). We also identified a considerable number of GO terms (30) that were overrepresented among the root-specific genes, including “apoplast” “plant-type cell wall”, “heme binding” and “response to desiccation” as the most overrepresented GO terms. Among all seven organ classes, the fruit-specific and seed-specific genes produced the lowest number of GO terms: the only GO term that was overrepresented for the fruit-specific genes was “chitin binding” while there were none associated with the seed-specific genes.

### Gene expression variation and conservation of gene expression

**Fig. 7:**
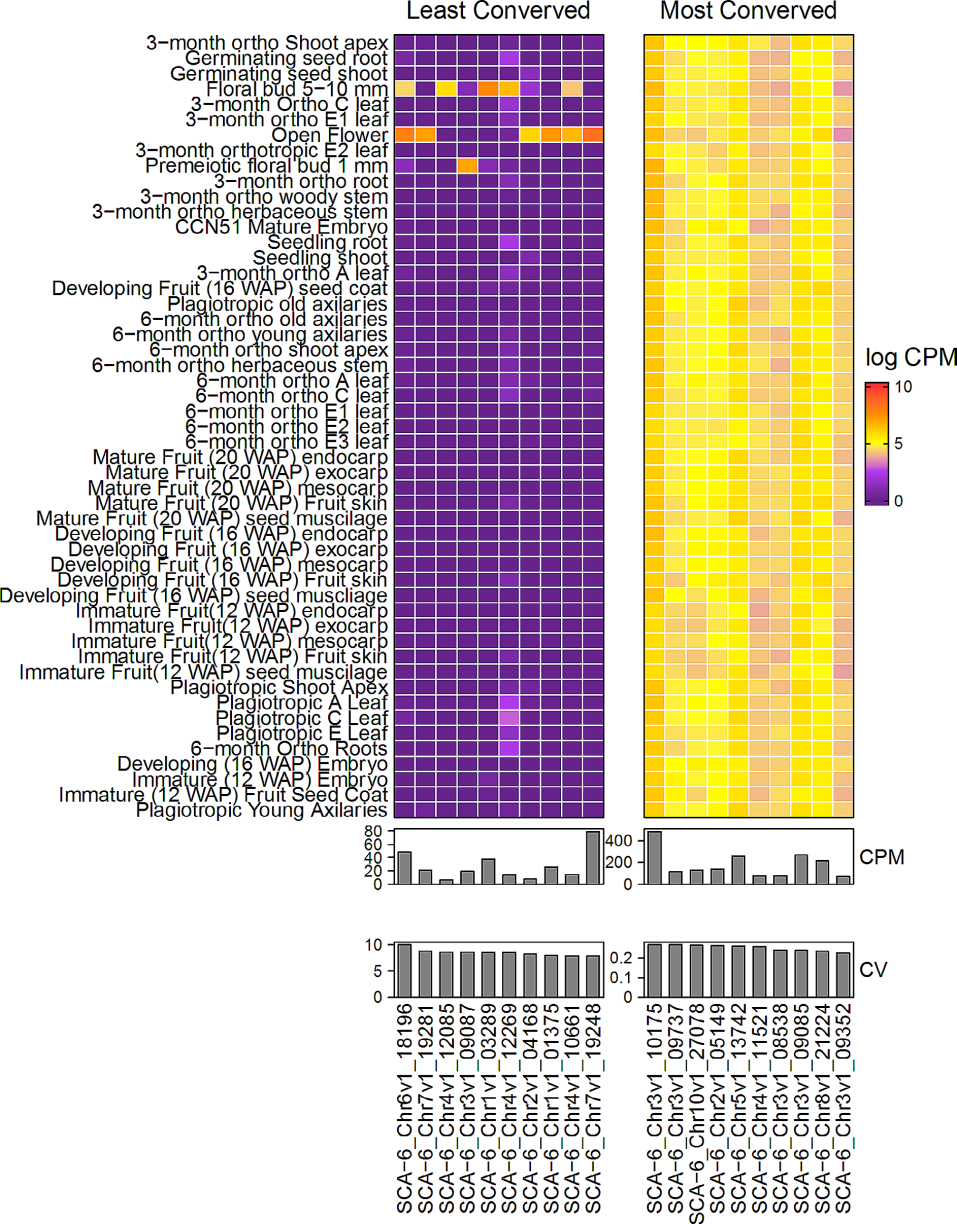
Heatmap of Most and Least Conserved *T. cacao* Genes by Coefficient of Variation (CV). Heatmap of 10 most and least conserved genes by CV across all replicates in the *T. cacao* Gene Atlas. Genes are plotted across the x-axis and tissue types are plotted across the y-axis. Expression values are plotted as log-transformed means for a respective tissue. Read counts were normalized by adding one read count to each value. Mean expression and CV for a respective gene across the atlas are plotted in bar plots underneath each heatmap. Heatmaps and bar plots were assembled using the *R* package *ComplexHeatmap*

Constitutive genes are expressed in all or most tissues in an organism, but the level of expression can vary by orders of magnitude during development and in different tissues. A subset of these genes is also very consistent in their expression levels, showing a low coefficient of variation among the different tissues and developmental stages. To explore the constitutive genes of cacao, we performed an analysis on our Gene Atlas dataset to identify genes that were consistently expressed with low coefficients of variation in all the samples we sequenced. While genes such as actin and tubulin, known as “housekeeping genes,” are expressed in nearly all tissues, their expression levels have been observed to change during development or in response to external factors. Therefore, we wanted to investigate genes that not only expressed across tissue types but also had highly conserved levels of expression. To achieve this, we calculated the coefficient of variation (CV) for each gene expressed above 30 CPM across all tissue types in the developmental atlas (51 tissues and 229 samples, see Additional File 4A) to determine the variability in expression across all tissues. Among the 1% of genes with the lowest CV (121 genes), the genes with the lowest CV: include an *RNA-binding KH domain-containing protein RCF3***(**SCA-6_Chr3v1_09352), a *FYVEdomain protein* (SCA-6_Chr8v1_21224), *DNA-directed RNA polymerases II, IV and V subunit 6 A***(**SCA-6_Chr3v1_09085), a protein *Dr1 homolog***(**SCA-6_Chr3v1_08538), and *SNF1-related protein kinase catalytic subunit alpha KIN10***(**SCA-6_Chr4v1_11521) (Fig. 7; Additional File 4B). Among the genes with the highest variability of expression between tissues, we identified a non-specific lipid-transfer protein (SCA-6_Chr1v1_03289), a *MEN-8protein* (SCA-6_Chr3v1_09087), *egg cell-secreted protein 1.2* (SCA-6_Chr4v1_12085), *probable fatty acyl-CoA reductase 4* (SCA-6_Chr7v1_19248), *MYB-related protein 305* (SCA-6_Chr4v1_10661), and a gene of unknown function (SCA-6_Chr6v1_18196) (Fig. 7; Additional File 4C).

We found a majority of genes (61%, or 7,385 genes) displayed little variability in relative expression, as their coefficient of variation (CV) fell within one standard deviation of the mean CV for all 12,112 genes analyzed (Additional File 5A). However, we did not observe any correlation (R^2^ = 0.12, *p* < 0.05) between a gene’s expression level and its variability of expression across tissues when we plotted the CV of each gene expressed above 30 CPM against its mean expression in all tissues (Additional File 5B). To investigate whether there were any relationships between variability of expression, expression level, and specific molecular functions, we performed GO enrichment analysis on subsets of genes. When we looked at the 1% least variably expressed genes, we found an over-representation of GO terms such as “protein transport” (*P* = 2.64E-6; FDR = 0.004), “clathrin coat assembly” (*P* = 1.57E-5; FDR = 0.014), “glutamatergic synapse” (*P* = 2.16E-5; FDR = 0.014), “PcG protein complex” (*P* = 3.27E-5; FDR = 0.014), and “protein domain specific binding” (*P* = 3.98E-5; FDR = 0.014) (Additional File 4C). The 1% most variably expressed genes were over-represented for GO terms including “wax biosynthesis process” (*P* = 2.19E-13; FDR = 2.99E-10), “suberin biosynthetic process” (*P* = 2.12E-8; FDR = 1.45E-5), “pollen exine formation” (*P* = 7.69E-7; FDR = 3.5E-4) “very long-chain fatty-acyl-CoA metabolic process” (*P* = 9.52E-7; FDR = 3.72E-4), “very long-chain fatty acid biosynthetic process” (*P* = 2.34E-5; FDR = 0.008), and “anther wall tapetum development” (*P* = 5.15E-5; FDR = 0.016) (Additional File 4C).

Next, we took the 50 highest and lowest expressed genes for the 1% most and least variably expressed genes and performed a GO enrichment analysis. Genes with high expression levels and low expression level variability were over-represented for terms such as “MAPK cascade” (*P* = 1.31E-6; FDR = 0.002), “centrosome” (*P* = 5.84E-5; FDR = 0.042), and “PcG protein complex” (*P* = 6.12E-5; FDR = 0.042) while highly constitutive genes expressed at low levels were not over-represented for any GO terms at the 0.05 FDR threshold (Additional File 4D). Among the genes on the opposite end of the distribution (1% of genes with most variable expression), the lowest expressed genes were over-represented for terms such as “wax biosynthesis process” (*P* = 1.01E-7; FDR = 1.38E-4), “suberin biosynthesis process” (*P* = 1.46E-6; FDR = 1E-3), and “long-chain fatty-acyl-CoA metabolic process” (*P* = 6.12E-5; FDR = 0.033) and “floral whorl development” (*P* = 6.12E-5; FDR = 0.033) while the only GO term associated with most highly expressed was “wax biosynthetic process” (*P* = 3.18E-6; FDR = 0.004). While “wax biosynthetic process” was identified as overrepresented among both the highest and lowest expressed genes, different genes belonged to each bin.

## Discussion

The main goal of developing The Cacao Gene Atlas was to accelerate cacao genomics research, both for basic scientific exploration and to use for more efficient breeding of improved cacao varieties to support sustainable farming systems. The Atlas includes data on 28,287 genes and 123 different samples, resulting in 13,479,301 gene expression data points. The raw sequencing data is publicly and freely available on NIH-NCBI in the SRA archives (https://www.ncbi.nlm.nih.gov/bioproject/936437); the raw sequencing reads can be found under Accession No. PRJNA933172, the transcriptome data can be found under Accession No. PRJNA931994, and the infection data can be found under Accession No. PRJNA476877. The gene expression mapping matrices and all sample metadata are also available in Additional File 6. To make the data accessible to scientists with limited bioinformatic resources it is also accessible on the BAR as a Cacao eFP Browser via standard web browsers (see Methods). While transcriptome atlases exist for multiple plant species, they are mainly in model or crop organisms such as Arabidopsis, maize, soybean, and poplar, due to sequencing being a significant rate-limiting step when these atlases were developed [22, 25, 41, 42]. This resulted in a lack of genomic resources for “orphan crops,” which are crops not usually traded internationally, limiting resources allocated towards their research [43–45]. However, the recent proliferation of next-generation sequencing has enabled sequencing to become cheaper and easier at larger scales [46, 47] which has enabled the development of genomics tools via sequencing for non-model species including barley, strawberry, *Camelina sativa* [48–53] and cacao [17–19]. Our use of a targeted sequencing strategy (QuantSeq) and Nextgen sequencing technologies further reduced the cost per sample significantly and provided additional evidence that 3’ end sequencing produces high quality sequences comparable to other Nextgen technologies.

To validate the atlas, we compared the expression patterns of six cacao genes (*ACT7, LEA19, AGL16, TIP13, LHY, MYB2)* orthologous to known, conserved functions in Arabidopsis (Figs. 3 and 4) [29–33]. We found to a large degree, the cacao genes exhibited similar expression patterns to their orthologs in Arabidopsis, with some exceptions of divergence in expression patterns. For example, the cacao gene *TcACT7* was expressed in all tissues and had a similar expression profile in Arabidopsis. *TcLHY* followed a similarly expression profile as its Arabidopsis ortholog; expression was highest in leaf tissues and was most highly expressed during the dawn. *TcLEA19* and *TcAGL16* were highly expressed in mature embryos and floral tissues, respectively. In Arabidopsis, *LEA19* is expressed later in development in mature tissues while *AGL16* was expressed in roots, stems, and mature leaves, respectively. We also saw several genes with slight deviations from known expression profiles in Arabidopsis. *TcTIP13* was expressed in roots and flowers whereas its ortholog is predominately expressed in flowers and pollen in Arabidopsis. Finally, *TcMYB2* had highest expression in roots while in Arabidopsis it was highest in leaves during drought stress and roots during salinity stress. These data validate the integrity of our dataset and demonstrate that for these genes, expression patterns are largely conserved between cacao and Arabidopsis.

We also performed several global analyses on the developmental transcriptomes to better understand gene expression profiles and gene expression tissue specificity in cacao. We found the mean number of genes expressed across all tissue types was 5855 (20.6% of all genes in the genome); we also found the largest number of expressed genes among the plagiotropic old axillary buds (7816 genes) and the fewest in the open flower (4289 genes) (Table 2). This phenomenon contrasts those observed in other crop plants: in previously published Arabidopsis and *Camelina sativa* atlases, floral and seed tissues were those observed with the highest counts of expressed genes while leaf tissues had the fewest numbers of expressed genes [52, 54]. We postulate this might be an effect of the physiology of the respective plants. Cacao is a perennial tree crop grown in warm climates while Arabidopsis and *Camelina sativa* are annual crops grown in cooler climates. The starkly different climates these crops grow in, and their contrasting life cycles might require different allocations of resources for gene expression. Annuals are believed to have higher reproductive effort than perennials which has been shown to reflect differences in genetics and physiology [55, 56]. We also see this phenomenon supported in other organisms such as maize and *Lotus japonicus* where flowers are among the tissues with the most tissue-specific genes [57].

We established two methods of classifying a gene as tissue-specific: with a row means filter to identify “functionally” tissue-specific genes and with a read count method to identify “extremely” tissue-specific genes. With each analysis we found the most tissue-specific genes among the immature embryo, open flower, and 6-month-old orthotropic E3 leaf (Figs. 5 and 6). This observation was similar to gene expression profiles observed in transcriptome atlases in other species: the leaf was identified as the maize organ with the largest number of tissue-specific genes while the flower and early developing seeds in *Camelina sativa* were identified as organs with the largest number of tissue-specific genes [52, 58]. We also performed the same analysis after binning the tissues into one of seven organ classes (axillary, root, shoot, stem, seed, fruit, flower). Here, we found the largest number of “extremely” and “functionally” organ-specific genes was in the cacao leaf (Additional File 3). Our investigation of GO terms of these tissue-specific genes supported our findings; leaf-specific genes were mostly associated with photosynthesis and chloroplast function, while root-specific genes were associated with stress response and carbohydrate metabolism (Additional File 3).

We also investigated the phenomena of gene expression across tissues in the cacao atlas to better understand how patterns of gene expression change across tissue types. After ranking genes by their coefficient of variation (CV) using mean expression, we performed a GO enrichment on the top and bottom 1% of genes found on this list. Among the 1% of genes with the lowest CV, we found an overrepresentation for genes involved in protein functions (vesicle-mediated transport, protein transport, regulation of protein metabolic processes, and intracellular transport) while genes associated with the 1% of most variably expressed genes were overrepresented for terms associated with wax and fatty-acid biosynthesis (wax biosynthesis, suberin biosynthesis, very long chain fatty acid biosynthesis, regulation of fertilization, cutin biosynthesis) (Fig. 7; Additional File 4). These variably expressed genes were mainly found to be expressed in floral tissues at the three stages of development that were sampled for the atlas (Fig. 7). Using a best reciprocal BLAST search to identify Arabidopsis orthologs of some of these variably expressed genes, we further identified their functions (Table 3). The *MEN-8* protein (SCA-6_Chr3v1_09087) was found to be an ortholog of an Arabidopsis lipid transfer protein found to be expressed in the anther tapetum, with later expression in the pollen exine during flower bud development [59]. Expression of this gene in cacao pre-meiotic floral buds indicates a similar role of this gene in cacao (Fig. 7). The *MYB*-related protein 305 (SCA-6_Chr4v1_10661) in cacao was found to be orthologous to *MBY21* in Arabidopsis. Expression of *MYB21* in Arabidopsis, along with other MYB genes is required for stamen elongation and development and is expressed in the anther and junction between the anther and stamen [60]. In cacao, this gene is found expressed in the developing bud and open flower tissue, again indicating a similar role for this gene between cacao and Arabidopsis (Fig. 7). A *probable fatty acyl-CoA reductase 4* (*FAR4*, SCA-6_Chr7v1_19248) was identified in cacao with the Arabidopsis ortholog also identified as *FAR4*. In Arabidopsis, *FAR4* expression was significantly downregulated in abscission mutants (*haehsl2*). As *FAR4* is one of the genes responsible for suberin biosynthesis, it is purported to play an important role in creating a suberin layer over the abscission zone after the floral tissue falls off the plant [60]. In cacao, *FAR4* is found to be expressed only in open flowers (Fig. 7). As cacao flowers are only open for about 24–36 h until they abscise, expression of *FAR4* in this tissue may be in preparation of floral abscission [61]. An understanding of gene expression patterns across our atlas might assist in answering other questions related to gene expression in cacao and other plant species. Considering the phenomenon of genes with tissue-specific expression is observed [62–64], further investigation into the diversity and mechanisms of tissue-specific genes in cacao might provide breakthroughs for understanding what mechanisms drive variability in gene expression between tissues and how this phenomenon works [65]. An understanding of tissue-specificity in cacao might lead to the acceleration of breeding for the tissue-specific traits that are economically valuable to farmers and breeders.

**Table 3:** Arabidopsis orthologs of cacao variably expressed genes

Cacao Gene ID	Arabidopsis Gene ID	Arabidopsis Gene Description (TAIR)	Cacao Annotation
SCA-6_Chr3v1_09087	AT5G52160	Bifunctional inhibitor/lipid-transfer protein/seed storage 2 S albumin superfamily protein	MEN8_SILLARecName: Full = Protein MEN-8; Flags: Precursor
SCA-6_Chr4v1_10661	AT3G27810	Encodes a member of the *R2R3-MYB* transcription factor gene family. Induced by jasmonate. Involved in jasmonate response during stamen development. *MYB21* interacts with *JAZ* proteins, and functions redundantly with *MYB24* and *MYB57* to regulate stamen development.	MYB05_ANTMARecName: Full = Myb-related protein 305
SCA-6_Chr7v1_19248	AT3G44540	Encodes a member of the eight-member gene family encoding alcohol-forming fatty acyl-CoA reductases (*FARs*) identified in Arabidopsis thaliana.	FACR4_ARATHRecName: Full = Probable fatty acyl-CoA reductase 4

Plant transformation could also benefit from understanding gene expression and tissue specificity. Many abiotic and biotic stress-responsive genes, which are of interest to plant breeders are expressed in a dose-dependent or copy number-dependent manner [66, 67]. The interest in utilizing these stress-responsive genes could spur interest in using high-expressing constitutive promoters such as cauliflower mosaic virus 35S or others [68] to achieve high levels of gene expression [69]. However, the utilization of these non-native promoters can lead to issues crop growth and development, sterility, enhanced pathogen susceptibility, among other issues that might impact yield and viability [70–72] as well as regulatory and public acceptance. It is therefore most beneficial for breeders to have access to native and/or inducible promoters that can better control transgene expression [73]. While there have been advances in stable and transient expression in cacao, there is a dearth of native and inducible promoters that might optimize plant transformation. Our atlas provides breeders and researchers with a breadth and depth of gene expression data which might better equip them to build a toolbox of transcriptional regulatory elements such as promoters, introns and terminators for cacao to optimize breeding [68].

An understanding of the variation in gene expression in a species has been performed in other plant species and could also improve an understanding of gene expression in cacao [74–78]. The identification of housekeeping genes for experiments such as qRT-PCR were originally selected in the “pre-genomics” era because of their roles in core cellular functions in the plant and the assumption expression of these genes across tissue types was ubiquitous [79, 80]. However, sampling from a single cultivar and other factors are all potential limitations of the validity of these previously selected housekeeping genes [78]. An analysis of gene expression of developmental Arabidopsis tissue showed tissue expression dynamics were not conserved between tissue types; leaf tissues were most resemblant of the profile of the entire dataset while root, flower, and pollen tissues were among those with wide ranges of expression compared to the average of all tissues [64]. Additionally, the assumption that these housekeeping genes are all expressed at the same level has been challenged in multiple plant species to improve housekeeping gene selection. Czechowski et al. [80] found several traditional housekeeping genes used in Arabidopsis were not among the most stable in the developmental atlas and polyubiquitin family genes along with genes coding for regulatory function proteins were identified as the most stable across the dataset. A similar analysis was performed in apple which resulted in the discovery of several candidate genes with lower variability between tissues including *LIPID TRANSPORT LIKE 1*, a *phytochrome-associated protein phosphatase 3* (*FYPP3*), and a *CK2 regulatory subunit* (*CKB4*) [78]. These data point to the potential of using gene expression CV to better understand gene expression across tissue types and gene families.

## Conclusion

The construction of a *Theobroma cacao* transcriptome atlas is a significant development for cacao, tropical crops, and tree genomics. The breadth and depth of genetic information available to breeders and researchers should enable considerable advances in cacao genomics. The establishment of this atlas also provides the opportunity to further contribute to the atlas through sequencing of tissues under a/biotic stressors, from a variety of genetic backgrounds, and under different environmental conditions. The identification of two classes of tissue-specific genes (extreme and functional) has also spurred questions about gene expression in cacao and what determines tissue-specificity across tissue types in a plant. Work has been initiated in this field of genomics with some understanding that promoter architecture, chromosome replication, and intron size all play roles in determining tissue specificity of a gene. However, the identification of tissue-specific genes across a wide breadth of tissues in cacao coupled with modern chromatin accessibility experiments (ATAC-seq) might contribute significantly to understanding tissue-specific gene expression in cacao with the potential of engineering tissue-specific promotors to accelerate transformation and breeding efforts.

We invite collaborators to submit additional data to the resource. In addition to supporting research into plant developmental biology, we hope this resource provides support for the cacao research community to accelerate the development of improved cacao varieties in support of farmers, the chocolate industry and towards more sustainable cacao farming systems.

## Materials and methods

The Cacao Gene Atlas comprises final datasets for six experimentally designed transcriptomic atlases: developmental, drought and diurnal, seed, meristem, leaf, and leaf infection. The leaf infection atlas is derived from data previously reported [26]. Materials and methods for the experimentation, data generation, and analyses of the remaining five atlases are described below.

### Plant Source Material and Growth conditions

Cacao fruits, genotype CCN-51, at twelve-, sixteen-, and twenty-weeks after pollination (WAP), were used as source materials for the developmental, drought and diurnal, and seed atlases. The fruits were harvested from open-pollinated, mature, clonally propagated trees by Fernando Crespo on Rancho San Jacinto, Naranjal, Ecuador. Fruits were cleaned, packaged, and shipped to The Pennsylvania State University (University Park, PA) for sample collection, planting, and further molecular work. Material from three fruit developmental stages was collected and seeds from 20-WAP fruits were planted as source material for later tissue collections. The fruits were split open using a rubber mallet and seeds were removed. Seed coats were scored with a razor blade and removed by hand. The seeds were then soaked in room temperature water for seven hours and sown in well-watered, autoclave-sterilized 1:1 peat: perlite mixture (ProMix BX Growing Medium and Whittemore Super Corse Graded Horticultural Perlite) (1020 tray/ 32 cell insert) to an approximate depth of half the seed length (~ 2.5 cm) with the hypocotyl facing down. The trays with seeds were incubated at greenhouse conditions as described below (University Park, PA, Tyson Building Greenhouse J). To maintain high humidity in the initial stage, the trays with the seeds were covered with humidity domes. Following germination and the expansion of true leaves, humidity domes were removed, and plants were bottom-watered as needed with 2 L/tray Hoagland’s Solution [81]. Plants were re-potted twice as plants aged: (1) seedlings 60-days past germination (DPG) were transplanted into 4-inch Azalea plastic green pots in a perlite:sand:Turface mixture (4:2:1) (All Sport Pro Turface, Lesco) and placed on automatic drip irrigation of Hoagland’s Solution, and (2) at 210-DPG (7-month old), plants were transplanted again into 5-gallon Nursery pots using perlite:sand:Turface (4:2:1) (All Sport Pro Turface, Lesco). When moved to automatic drip irrigation (2 L/hour), plants were watered daily at 8:00 AM, 12:00 PM and 6:00 PM for 6, 2, and 2 min, respectively. The greenhouse conditions were 80–85 °F, 60–65% humidity, 16-hour photoperiod supplemented with LED lights.

In addition to CCN-51 material, mature fruits from genotypes IMC67 and SCA6 were sourced from at USDA ARS Tropical Crops and Germplasm Research fields (Mayaguez, Puerto Rico) used for the seed atlas. Mature fruits were shipped in mesh bags packed in cardboard boxes. Embryos were collected on arrival at The Pennsylvania State University.

To eliminate potential gene expression variation that might result from segregating CCN-51 seedlings, for the meristem and leaf atlases samples were collected from SCA6 and ICS1 plants clonally propagated at The Pennsylvania State University greenhouse. Plants were propagated by rooted cuttings and grown as previously reported [81].

All the data presented in this manuscript should be interpreted with the understanding of the plant growth conditions detailed above. These results are only relevant to these conditions and gene expression levels could differ under different growth conditions.

### Tissue Collection

Artist renditions of various organs and tissues of the cacao tree throughout its lifecycle are presented in Figs. 1 and 2 and descriptions of the various stages of cacao development are also included in the results section. Additionally, detailed descriptions of all samples collected are included in Additional File 6. Samples were harvested and immediately flash-frozen in liquid nitrogen then transferred to temporary storage at -80 °C. All samples were collected in replicates of 3–5, and some samples were from pooled tissues from multiple trees as detailed below and in Additional File 6.

### Description of the tissues sampled

All metadata describing sample collections are listed in Additional File 6. Cacao fruit/fruit (skin, exocarp, mesocarp, endocarp) and seed (mucilage, coat, embryo) tissues were collected immediately after arrival at Penn State from three stages of fruit development (Additional File 6). CCN-51 seedlings were grown in the greenhouse (as described above) and the following samples were harvested: germinating seed tissue (closed cotyledon stage shoot and root systems harvested separately), seedling tissue (~ 30-DPG, shoot and root systems harvested separately), 90-DPG (3-month-old) and 180-DPG (6-month-old) orthotropic plant tissues including root (30 cm of root system above root tip), leaf (developmental stages A, C and E as defined in [82]), shoot apex (1 cm) and stem (herbaceous section – 2 cm of tissue below shoot apex; woody section − 2 cm of tissue above cotyledons). 180-DPG seedlings were also the source of young and old orthotropic apical buds, harvested from axils of the youngest leaves and oldest leaves on the tree.

To evaluate differences in gene expression between young seedlings and mature plants, tissues were collected from 1.5-year-old plagiotropic plant, which included leaves (developmental stages A, C and E as defined in [82]), shoot apices (1 cm), old and young (woody and herbaceous) axillary buds. Floral tissue was also collected from 1.5-year-old trees, including premeiotic buds (smaller than 1 cm), floral buds (5–10 cm), and open flowers. All samples were collected at the same time of day (11AM – 1 PM).

Tissues from 8-month-old, greenhouse-grown CCN-51 seedlings were collected for the drought and diurnal atlas. Well-watered plants were grown as described above, whereas drought-stressed plants were taken off irrigation for two days prior to tissue collection. Orthotropic apices, leaves at developmental stage E, and 10 cm of the lateral root, including root tips, were sampled for this experiment every four hours over 24 h period, starting at 5 AM.

Much of the atlas is composed of a segregating population of open-pollinated CCN-51 progeny growing in a monoculture field of CCN-51 trees; however, the atlas also contains samples from genotypes other than the CCN-51 cultivar to focus on several important cacao features and responses [83]. Samples were harvested from clonally propagated SCA6 plants to study meristem development. The stages of leaf development in cacao were defined previously (stages A, B,C, D and E) [84]. We divided the leaf-flush cycle (described below) into four stages for sample collection [12]. Orthotropic and plagiotropic shoots where sampled in replicates as follows: Stage 1 indicates an early-stage active apex with the youngest leaves being small A leaves (smaller than 2 cm in length), Stage 2 indicates a later-stage active apex with the youngest leaves being larger A leaves (ranging from 0.5 to 10 cm), Stage 3 indicates an early dormant apex with the comprising of C and D leaves, and Stage 4 indicates a late dormant apex comprised of E leaves. Stages 1–4 are depicted at http://bar.utoronto.ca/~asher/efp_cacao_sca/cgi-bin/efpWeb.cgi.

To investigate the influence of genotype on gene expression during seed development, we developed a seed atlas consisting of CCN-51, IMC67, and SCA6 embryos sourced from open-pollinated mature fruits. Additionally, we created a leaf development atlas that includes samples from stages A - E as defined in Mejía et al. [82] from clonally propagated SCA6 and ICS1 genotypes. Finally, previously reported data [20, 26] was incorporated to create leaf infection atlas representing genotypic differences in gene expression in response to pathogen infection. This atlas contains Stage C leaves from clonally propagated NA32 and SCA6 treated with either with *P. megakarya* or a control water infiltration as previously reported [20, 26].

### Tissue Processing

Frozen tissues were ground using one of two protocols as described in Additional File 6. Most samples were processed into a fine powder using a SPEX 6875D Freezer/Mill® Dual Chamber Cryogenic Grinder (SPEX Sample Prep, Metuchen, NJ, USA). Some samples were ground manually using a mortar and pestle with liquid nitrogen until the tissue was ground into a fine powder. Approximately 100 mg of the powder was then aliquoted into 2 ml screw cap tubes for RNA extraction and the remaining ground tissue was stored at -80 °C.

### RNA extraction

RNA was extracted from 100 mg of homogenized tissue using 1 mL extraction buffer (1% IGEPAL, 100mM EDTA, 0.02% SDS, 20% b-mercaptoethanol, 0.5% sodium azide). Most samples had 100 mg of tissue aliquoted; samples that were very sticky (stage A leaves, stage C leaves, apices and axillary buds) had less tissue aliquoted (approximately 20–40 mg). Samples were vortexed until tissue was homogenized in the buffer then centrifuged at 16,000 x g at 4 °C for 20 min. 200 µl of 5 M NaCl and 600 µl of chloroform was added to the eluant; the eluant was centrifuged for 5 min at 16,000 x g at 4 °C. Chloroform extraction was repeated twice using an equal volume (1:1) of chloroform to the organic extraction aqueous layer. After organic extraction, an equal volume of room temperature isopropanol was added to the aqueous layer of the organic extraction and incubated at room temperature for 10 min, then centrifuged using parameters described above. Isopropanol was then removed, and the remaining pellet was washed with 1 mL of 70% ethanol and centrifuged using the same parameters as above. The supernatant was removed, and the ethanol wash was repeated two more times. Finally, RNA pellets were allowed to dry for approximately 10–20 min before resuspension in 20–40 µL molecular grade water (VWRL0201-0500; VWR; Radnor, PA). RNA samples that did not pass purity checks (described below) but had intact RNA were further purified by ethanol precipitation [85]. Detailed RNA extraction protocol is described in Additional File 7 to be used as a resource for cacao RNA extractions.

One µL of each extracted RNA was analyzed on a Nanodrop 2000c (Thermo Scientific, Waltham, MA, USA) for concentration and purity based on 260/230 and 260/280 ratios. RNAs were considered to pass quality control if their concentrations and volumes yielded at least 3 µg of total RNA and their 260/280 ratios were at least 1.8. In addition, RNA quality was analyzed by agarose gel electrophoresis. 1 µl of RNA, 8 µl of nuclease free water, and 2 µl 6X loading dye were mixed and loaded into a well of a 1.5% agarose gel alongside 1 kb DNA ladder (New England Biolabs, Ipswich, MA, USA). The gel was analyzed for intact 28S and 18S ribosomal RNA bands. In the event a sample was very sticky, had visible bands at 28S and 18S and smearing on the gel that resembled degradation, the sample was cleaned up using the protocol below and then re-evaluated with a nanodrop and gel.

### DNase treatment and column purification

Three µg of RNA were further purified to remove residual contaminating DNA using DNaseI, RNase-free (Thermo Scientific, catalog #EN0521) according to manufacturer’s protocol (Publication no. MAN0012000). Post-DNase treatment, RNA samples were further column purified using RNA Clean and Concentrator-5 kit (Catalog # R1013, Zymogen Research, Irvine, CA, USA). Modification of manufacturer’s instructions included final elution of floral sample RNAs in 15 µl of 55 °C RNase-free water and a 5-minute incubation for the final elute before the final centrifugation step.

### Post-treatment Quality Control

Following DNase treatment and column purification, RNA samples were again assessed for quantity and quality using the Agilent 4200 Tapestation System (Agilent Technologies, Santa Clara, CA, USA) at The Pennsylvania State University Huck Institute’s Genomics Core Facility using 3 µL of RNA. Integrity Number (RIN) and percentage of RNA fragments above 200 nucleotides (DV200) were recorded for each sample. Samples with RIN values between 4 and 10, and RNA concentration above 20 ng/mL were used for library construction. Samples were diluted to 50 ng/ul in water for library production. Several samples had lower starting yields due to sample limitations (Additional File 8).

### Preparation of cDNA libraries and sequencing (RNA-Seq)

For the developmental, drought and diurnal, meristem, seed, and leaf infection atlases, preparation of libraries and sequencing was conducted at the Penn State Genomics Core. The libraries construction and sequencing of the leaf development atlas had was performed at the Oregon State University Center for Genome Research and Biocomputing. The developmental, drought and diurnal, meristem, seed, and leaf development atlases had libraries prepared using a 3’ UTR specific sequencing method, (QuantSeq 3’ Lexogen GmbH, Vienna, Austria) while the Leaf Infection Atlas had libraries prepared using TruSeq (Illumina Inc. San Diego, CA, USA). QuantSeq produced single end reads, with 8 million reads per replicate with 75 bp reads. On average, reads mapped between 79 and 82% efficiency (Additional File 9). Most libraries in the atlas were sequenced using an Illumina HiSeq 2500, except for the Leaf Development Atlas which used the Illumina HiSeq 3000 platform.

### Transcriptome sequencing

The library construction employed a 3’ UTR-specific sequencing method called QuantSeq 3’ (Lexogen GmbH, Vienna, Austria), which generates reads near the 3’ end of the polyadenylated tail using the last exon and untranslated region to generate a single read per transcript [86]. This method is advantageous over randomly-primed cDNA sequencing methods like TruSeq (Illumina Inc. San Diego, CA, USA) due to significant cost reduction and requirement of fewer reads per sample to achieve the same level of sequencing coverage. QuantSeq 3’ sequencing has been found to produce high quality and reproducible data, particularly in organisms with high quality genomes [87–89]. In a pilot experiment, we tested the correlation of gene expression measurements using identical RNA purified samples used to produce both QuantSeq and TruSeq libraries, which were then sequenced identically. A Pearson correlation of DESeq2 regularized log-transformed counts for each method indicates a strong positive correlation to each other (R^2^ = 0.97, Additional File 10); the correlation was strong even when comparing 3 M QuantSeq reads to 30 M TruSeq reads (Additional File 10). Based on this data, we sequenced the atlas samples with a target of eight million reads per sample, as opposed to 25 million reads per sample we used for TruSeq, providing a 3-fold reduction in sequencing costs with no measurable loss in data quality. The libraries were also less expensive to produce compared to TruSeq, resulting in a total reduction in price of about eightfold. Using QuantSeq sequencing allowed us to sample and sequence 390 libraries for the cacao atlas due to the lower cost of sequencing, allowing us to create a more comprehensive atlas. Expression levels of all detected genes ranged from our cutoff (1 CPM), below our threshold of what we consider background (30 CPM) to the strongest expressed gene (21,614 CPM).

### QuantSeq RNA-seq read Processing

Raw QuantSeq reads were first examined with FASTQC (v0.11.9 https://www.bioinformatics.babraham.ac.uk/projects/fastqc/) to assess the overall data quality before processing. Reads were then processed using *bbduk* (BBMap tools v37.76; https://jgi.doe.gov/data-and-tools/software-tools/bbtools/bb-tools-user-guide/bbduk-guide/) to trim the adapter sequences, poly-A tails, and low-quality bases and to discard fragments less than 20 bp in length after trimming. Trimmed reads were mapped to the CCN-51 and SCA6 *Theobroma cacao* genotype reference genomes using the STAR Aligner version 2.7.5b [90]. Expression quantification was performed with *featureCounts* from the Subread package version 2.0.1 [91] in a fractional read-counting mode to prop distribute muti-mapping reads among features using gene annotation GFF3 files modified with GenomeTools version 1.5.9 [92] to include intron coordinates. The count matrices were normalized to counts per million (CPM) values using the default parameters of the *cpm* function in the *edgeR* Bioconductor package [93]. Annotations were performed as described in Winters et al. [20]. Gene accession numbers across genotypes and gene annotations can be found in Additional File 11. Complete functional annotation of gene sets was performed using the Blast2GO [94] functional annotation module. The best functional descriptors for gene products were assigned following BLASTp searches against the UniProt/SwissProt databases. Analysis commands utilized in the QuantSeq read processing are reported in Additional File 12. Leaf infection counts from Pokou et al. [26], CPM, and fractional read counts (10.5061/dryad.0k6djhb59) can be found in Additional File 6.

### Cacao eFP browsers

Images for the browser were generated using Adobe Illustrator (Adobe Inc., available at: https://adobe.com/products/illustrator) and GIMP (The GIMP Development Team, 2019. GIMP, available at: https://www.gimp.org). Appropriate parts of the images were filled with unique hues to correspond to specific samples described in this paper. Data as CPM values were transferred to the BAR server located at the University of Toronto [95]. Since each cacao genome assembly has a different annotation set, which give different gene numbers and accession IDs, to simplify the use of the BAR cacao resource, we created three browsers based on three reference genome assemblies (SCA6, CCN51, and Criollio). The SCA6 genome assembly was used to map expression levels in the developmental, drought and diurnal, meristem, and seed atlases (Cacao SCA eFP Browser), whereas the CCN-51 genome assembly was used for mapping the developmental and drought and diurnal atlases (Cacao CCN eFP Browser), and the Criollo assembly was used for mapping the leaf development and leaf infection atlas (Cacao TC eFP Browser). After image creation, XML configuration files for all three eFP Browsers, based on the genome assembly the reads are mapped on, were generated using a custom tool.

### Read mapping, normalization and threshold cutoffs

To compare gene expression across different tissue types in the developmental gene atlas, we used the fractionalized counts per million (CPM) data matrix. To determine if a gene was “expressed,” we required it to meet a read count threshold in at least two libraries for 3x replicated tissues, or at least three libraries for 4-5x replicated tissues. Next, we sought to determine tissue-specific (TS) expression among these expressed genes. Initially, we considered using a threshold of 1 CPM, but this would have included many genes expressed at low levels in all tissues, which could be considered background noise [96, 97]. Instead, we sought to identify an optimal read count cutoff that would eliminate the most background noise while identifying the most tissue-specific (TS) genes in the most libraries. We established that a cutoff of 30 CPM was reasonable as it identified the highest number of TS genes per library (21.9 genes per library). We defined two types of TS genes: “extremely” TS genes, which were expressed above 30 CPM in only one tissue type, and “functionally” TS genes, which were expressed above 30 CPM in a tissue and expressed more than two-fold compared to the mean of all other tissues combined [X _tissue_ > 2(X _all other tissues_)].

### Transcriptome analysis

To explore gene sets which are expressed in tightly controlled organ, tissue, or developmental time points, we established two definitions of specific gene expression. For this study, we defined tissue-specific genes as “extremely tissue-specific” (expression > than 30 CPM in only one sample), or as “functionally tissue-specific” (mean expression greater than 30 CPM in a tissue and twice the mean expression of all other samples). “Extremely organ-specific” genes were discovered by identifying genes expressed above 30 CPM in each tissue then pooling genes belonging to a particular organ class (axillary bud, flower, leaf, fruit, seed, root, or shoot). These pooled lists were then cross-checked against each other to find only unique genes belonging to each organ class. “Functionally organ-specific” genes were identified by pooling tissues of a particular organ class together, then calculating row means and comparing gene lists as was performed with the “functionally tissue-specific” analysis. One read count was added to each gene and tissue type in the matrix for normalization. Mean read counts and coefficient of variation (CV) between the replicates were computed using base R. GO term enrichment for tissue-specific genes was performed with OmicsBox Blast2GO and Enrichment Analysis packages using a Benjamini-Hochberg multiple test correction [94]. GOs were classified as overrepresented with an FDR filter of 0.05 and were reduced to more specific GO terms with an FDR filter of 0.05. The background gene list for the GO enrichment analysis was all genes expressed above 30 CPM in at least one tissue type. Heatmaps, bar plots, and scatterplots were assembled with the R-packages *ComplexHeatmap* and *ggplot2*, respectively [98, 99].

### Electronic supplementary material

Below is the link to the electronic supplementary material.

**Additional File 1:** Principal Component Analysis of Sample Replicates

**Additional File 2:** Tissue-specific Genes

**Additional File 3:** Organ-specific Genes

**Additional File 4:** Coefficient of Variation Data

**Additional File 5:** Histogram and Scatterplot of CV of Gene Expression in the T. cacao Gene Atlas

**Additional File 6:** Replicate Count Data and Metadata

**Additional File 7:** RNA Extraction and Clean Up Protocol

**Additional File 8:** RNA Quality Values

**Additional File 9:** Read Count Statistics

**Additional File 10:** Pearson Correlation of QuantSeq and TruSeq Counts

**Additional File 11:** Gene Annotations and RBBH

**Additional File 12:** Quantseq RNA-seq processing software commands and parameters

## Data Availability

The EFP browsers generated during the current study are currently available at the following links: The Cacao SCA eFP Browser is at https://bar.utoronto.ca/efp_cacao_sca/cgi-bin/efpWeb.cgi. The Cacao CCN eFP Browser is at https://bar.utoronto.ca/efp_cacao_ccn/cgi-bin/efpWeb.cgi. The Cacao TC eFP Browser is at https://bar.utoronto.ca/efp_cacao_tc/cgi-bin/efpWeb.cgi. The Gene Atlas is available at National Center for Biotechnology Information (NCBI) and the hyperlinks provided in the text. All data used to perform analyses is available in the additional files. The reads for this project are available through the NCBI Sequencing Read Archive (SRA) under the Bioproject Accession number PRJNA936437 and at the following link: https://www.ncbi.nlm.nih.gov/bioproject/?term=PRJNA936437. The Cacao Gene Atlas is listed under Sub-bioproject PRJNA933172 and is available here: https://www.ncbi.nlm.nih.gov/bioproject/933172. Biosamples sequenced for the Gene Atlas can be found from numbers SAMN33227142 to SAMN33227529. Sequencing reads for this project can be found at Singe Read Archive Accession Numbers SRR23422485 to SRR23422872. The reads for the leaf developmental atlas can be found under Bioproject PRJNA931194 under SRAs SRR23422980 to SRR23423009. Additional File 6 can be found at the following Dryad link: 10.5061/dryad.0k6djhb59.

## Abbreviations

NGS: next-generation sequencing
SNP: small nucleotide polymorphism
BAR: Bio-Analytic Resource for Plant Biology
UTR: untranslated region
cDNA: copy DNA
CPM: counts per million
eFP: electronic fluorescent pictograph
TS: tissue-specific
TC or Tc: *Theobroma cacao*
WAP: weeks after pollination
OS: organ-specific
GO: gene ontology
TF: transcription factor
CV: coefficient of variation
FDR: false discovery rate
Cd: cadmium
DPG: days past germination
RIN: RNA integrity number
Bp: base pairs
ZF: zinc finger
ABA: abscisic acid
q-RT-PCR: real-time quantitative reverse transcription polymerase chain reaction
ATAC-seq: assay for transposase-accessible chromatin using sequencing
