# Supplementary material for: The cacao gene atlas: a transcriptome developmental atlas reveals highly tissue-specific and dynamically-regulated gene networks in Theobroma cacao L
Source: BMC Plant Biol. 2024 Jun 26;24:601. doi: 10.1186/s12870-024-05171-9 (PMC11201900; doi:10.1186/s12870-024-05171-9)
Supplement: Supplementary file 1 — Additional File 1: Principal Component Analysis of Sample Replicates [file 12870_2024_5171_MOESM1_ESM.docx]

**
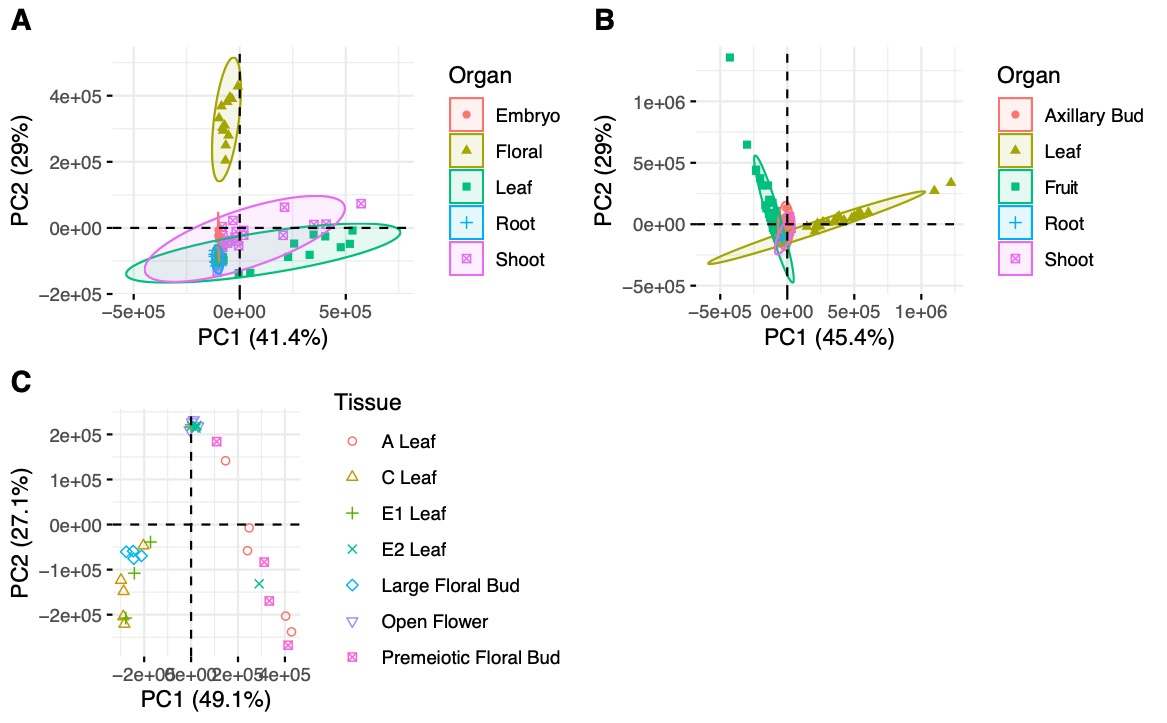
**

**Additional File 1.1**. Principal component analysis of sample replicates from Developmental Atlas using all genes represented in the atlas. (A) Samples in the developmental atlas corresponding to germinating seed, seedling, 3-month-old orthotropic plant, floral tissues, and mature embryo tissues. (B) Samples in the developmental atlas corresponding to 6-month-old orthotropic plant, 2-year-old plagiotropic plant, and fruit tissues. (C) Samples in the developmental atlas corresponding to leaf and floral tissues. Ellipses indicate 95% confidence intervals.

**Additional File 1.2**. Principal component analysis of fruit tissue sample replicates from Developmental Atlas using all genes represented in the atlas. (Left) samples grouped by age of tissue. (Right) samples grouped by tissue type.

**Additional File 1.3**. Principal component analysis of all Leaf tissue sample replicates from Developmental Atlas using all genes represented in the atlas. (Left) samples grouped by leaf stage. (Right) samples grouped by age of leaves.

**Additional File 1.4**. Principal component analysis of all tissue sample replicates from the Drought Diurnal Atlas using all genes represented in the atlas. (Left) samples grouped by organ. (Right) samples grouped by age of treatment. Ellipses indicate 95% confidence intervals.

**Additional File 1.5.** Principal component analysis of (Left) all samples from the Seed Atlas grouped by genotype, ellipses indicates 95% confidence interval. (Right) All samples from the Meristem Atlas grouped by growth stage.
