# Supplementary material for: The cacao gene atlas: a transcriptome developmental atlas reveals highly tissue-specific and dynamically-regulated gene networks in Theobroma cacao L
Source: BMC Plant Biol. 2024 Jun 26;24:601. doi: 10.1186/s12870-024-05171-9 (PMC11201900; doi:10.1186/s12870-024-05171-9)
Supplement: Supplementary file 5 — Additional File 5: Histogram and Scatterplot of CV of Gene Expression in the T. cacao Gene Atlas [file 12870_2024_5171_MOESM5_ESM.docx]

**Additional File 5. Histogram and Scatterplot of CV of Gene Expression in the *T. cacao* Gene Atlas. (A)** Histogram of the distribution of coefficient of variation (CV) of genes in the *T. cacao* gene atlas expressed above 25 CPM. X-axis represents the log-transformed CV, the left y-axis represents the number of genes, and the right y-axis represents the density of the histogram. The distribution curve is represented by the red line, the vertical black line represents the mean CV, and the dashed vertical lines represent one standard deviation (SD) above or below the mean CV. **(B)** Scatterplot of the CV for all genes expressed above 25 CPM plotted against their log-transformed mean expression for the gene across the atlas. The red line represents the regression line with corresponding r-squared and p-values shown.
