# Supplementary material for: The cacao gene atlas: a transcriptome developmental atlas reveals highly tissue-specific and dynamically-regulated gene networks in Theobroma cacao L
Source: BMC Plant Biol. 2024 Jun 26;24:601. doi: 10.1186/s12870-024-05171-9 (PMC11201900; doi:10.1186/s12870-024-05171-9)
Supplement: Supplementary file 6 — Additional File 6: Replicate Count Data and Metadata [file 12870_2024_5171_MOESM6_ESM.docx]

Additional File 6 can be found at the following Dryad link: https://doi.org/10.5061/dryad.0k6djhb59.
