# Supplementary material for: The cacao gene atlas: a transcriptome developmental atlas reveals highly tissue-specific and dynamically-regulated gene networks in Theobroma cacao L
Source: BMC Plant Biol. 2024 Jun 26;24:601. doi: 10.1186/s12870-024-05171-9 (PMC11201900; doi:10.1186/s12870-024-05171-9)
Supplement: Supplementary file 7 — Additional File 7: RNA Extraction and Clean Up Protocol [file 12870_2024_5171_MOESM7_ESM.docx]

**Additional File 7. RNA Extraction and Clean Up Protocol**

**Gene Atlas RNA Prep Protocol:**

**For both your protection and the integrity of your RNA, wear gloves throughout preparation. Change gloves if you encounter a surface that might have a high level of RNases (such as your face, hair or a door handle). Chloroform and liquid nitrogen (N^2^) are also used in this protocol, use of proper PPE is suggested.**

1. Sample harvest: Excise the tissue of interest from the plant using sterilized tweezers and/or scalpel and immediately place in liquid N^2^ in pre-chilled, labeled aluminum foil packets. Tissue should be flash-frozen immediately after harvest to prevent any RNA degradation or unwanted RNA expression not representative of the tissue. Tissue should be kept frozen at all times until the addition of extraction buffer (step 4).
2. Sample grinding: Samples can be ground either by hand or using a cryo mill.
   1. To grind by hand, prepare a clean mortar and pestle for each sample to prevent cross contamination. Wash mortars and pestles with a 10% Liquinox solution (Alconox; White Plains, NY) by scrubbing well and triple rinsing with RO water.  Treat the mortars and pestles with a 0.1N sodium hydroxide (NaOH) solution using a squeeze bottle and letting sit for 3 minutes.   Repeat triple rinse with DEPC-treated water. Make sure all NaOH is removed.  Cover mortars and pestle ends with aluminum foil and bake in the oven for 4 h at 200°C, allow to cool before use.
   2. Pre-chill mortars and pestles at 4°C. Once chilled, put additional liquid N^2^ is inside the mortar with the tissue. Grind with chilled pestle until a fine powder is reached, adding additional liquid N^2^ as needed if it begins to evaporate. Ground powder is then transferred to pre-chilled and labeled 5 mL tubes and stored at -80°C until RNA extraction.
   3. To grind using a cryo mill (SPEX 6875D Freezer/Mill® Dual Chamber Cryogenic Grinder (SPEX Sample Prep, Metuchen, NJ, USA)), place each sample into an appropriately sized, pre-chilled grinding vial then put into the mill. Grind for three 1-minute grinding cycles with a 1-minute cool-down between each cycle for a total of five minutes per sample.
3. Sample aliquoting: Remove vials of pre-ground samples from the -80°C to a vessel with liquid N^2^ to prevent degradation. Per sample, label a 2-mL screw cap tube with the sample serial number then set in liquid N^2^ bath. Chill a spatula in your liquid N^2^ bath, then use the spatula to transfer ground tissue into pre-chilled screw cap tube. Re-chill spatula as needed so tissue is not exposed to a warm spatula (clean spatula with 70% ethanol in between samples). Approximately 100 mg of ground tissue is added to each tube. **Reminder: It is important tissue remains frozen during this process.**
4. Addition of RNA buffer: Working 4 samples at a time, move tubes from liquid N^2^ bath to centrifuge rack in fume hood and quickly add 1 mL RNA extraction buffer (protocol for RNA extraction buffer at the end of the document). Vortex tubes until tissue is homogenized in buffer. Lay tubes on their side to increase surface area. Once all samples have incubated in buffer for 5 minutes at RT, centrifuge at 15,000 rpm, 4°C for 20 minutes in an Eppendorf centrifuge 5424 R (Eppendorf, Hamburg Germany). While samples are spinning, prepare a new set of 2 mL (snap cap) RNase-free tubes (label with serial number).
5. **Start working on ice here and until isopropanol precipitation.** After centrifugation, pour off supernatant into fresh 2 mL tubes. If supernatants are very viscous, split into two 2 mL tubes and add 500 μL of fresh plant reagent buffer to each. These samples will now need to be processed as two separate samples that can usually be recombined before isopropanol precipitation.
6. Add 200 μL of 5M NaCl, finger flick to mix.
7. **From here, RNA is no longer protected by buffer reagents, work cleanly.** Add 600 μL of chloroform. Mix well by inversion. Centrifuge for 5 minutes at 4°C and 15,000 rpm. Transfer 800 μL aqueous phase into a new, labeled 2 mL snap cap.
8. Repeat cholorform extraction 1 -2 times with equal volume chloroform each time (chloroform volume equal to the aqueous phase volume being extracted). For second extraction, use 800 μL of chloroform. Transfer 600 μL of aqueous phase from second extraction. In the last chloroform extraction, the interphase should be spottier rather than a solid white precipitate. Usually, the volume of aqueous layer reduces 100 – 200 μL with each extraction. Transfer final aqueous phase into a snap cap (RNase-free) 1.5 mL tube. Label tube with serial number on top and side. Also write ‘RNA’ and date on side of tube.
9. **Begin working at room temperature.** Precipitate RNA by adding an equal volume of isopropanol (usually this will be 600 μL after 2^nd^ chloroform extraction). Centrifuge for 10 minutes at 4°C and 15,000 rpm. Remove isopropanol supernatant from RNA pellet. If pellet is hard to see, you can leave behind ~50 μL of isopropanol.
10. Pellet washing. Add 1 mL 70 % EtOH (prepared with DEP-C water). Finger flick to dislodge pellet. Spin for 5 minutes at 4°C and 15,000 rpm. Remove EtOH being careful not to remove the pellet. Pellets should now be more visible in EtOH than they were in isopropanol.
11. Repeat EtOH wash (step 10) 2x times for a total of three washes. Air dry pellets in fume hood (preferably with sash down) until the inside of tubes are dry by visual inspection (inspect each tube).
12. Resuspend pellets in nuclease free water. Use 20 μL for most samples but can increase volume if sample are especially viscous. Resuspend the pellet by finger flicking, then using a mini-centrifuge, spin down briefly to move tube contents to bottom of tube. **Place tubes on ice.**
13. **QC 1:**Measure purity of RNA with a Nanodrop 1 ul of sample.
14. **QC 2:**Agarose gel electrophoresis: Prepare 1.5 % gel. Combine 1 μL sample with 4 μL nuclease free water and 1 μL of 6x loading dye. Run samples on gel in 1x TAE buffer alongside 5 μL1 kb ladder and 6x purple gel loading dye (NEB) at 85 volts. Run until good separation of 28S and 18S ribosomal bands.
15. **Storage.** If samples pass QC, check your labeling and store RNA at -80°C.
16. Samples are now ready to further purify with DNAse treatment and a final column clean up.

**Protocol for DNase Treatment of Gene Atlas RNA Samples:**

Product: Thermo Fisher DNaseI, RNase-free (catalog #EN0521)

This protocol is a scaled-up version of the manufacturer’s recommended protocol.

1. Calculations for DNase reactions are based on nanodrop quantification of extracted RNA. Water was added to each RNA sample (3μg) to bring the volume up to 24 μL of RNA based on the concentration measured using the nanodrop.
2. Pipette calculated water into RNase-free 1.5 mL tubes.
3. On ice, prepare a master mix of buffer and enzyme in RNase-free tube (this is for 18 samples, if doing a different amount adjust this accordingly):

10x reaction buffer with MgCl2      58.0 μL

DNase I, RNase-free                     58.0 μL

Pipette to mix. Use 125 μL repeat pipettor to add 6 μL master mix to RNA in tubes.

1. Add calculated RNA volumes to tubes (final reaction volume should be 30 μL). Finger flick to mix and quick spin.
2. Incubate reactions at 37 °C for 30 minutes. Return reaction immediately to ice.

**NOTE:** the DNase-treated RNA samples are stable on ice. This is a good place to take a break if needed before proceeding to column clean.

**RNA Column Clean Protocol:**

Product: Zymo Research RNA Clean and Concentrator-5 (Catalog Nos. R1013, R1014, R1015, R1016)

Protocol follows the manufacturer’s protocol with some adjustments.

Before processing, adjust the sample volume to 50 μL minimum (20 uL H_2_O added)

1. Add 100 μL RNA Binding Buffer to 50 μL samples. Mix thoroughly.
2. Add 1 volume (150uL to a 150uL mixture) 100% ethanol.  Mix thoroughly and transfer into a Zymo-Spin IC Column in a Collection Tube. Centrifuge at 15,000 rpm for 30 seconds at room temperature.
3. Add 400 uL RNA Prep Buffer to the column and centrifuge as above. Discard the flow through.
4. Add 700 uL RNA Wash Buffer to the column and centrifuge at 15,000 rpm for 4 minutes at room temperature to ensure complete removal of the wash buffer. Transfer the columns carefully into an RNase-free tube.
5. Add 15 uL DNase/RNase-Free Water directly to the column matrix, let sit for 1 minute, and centrifuge at 15,000 rpm for 30 seconds at RT.

- If samples are “sticky” and have failed to elute from the matrix , heat the water to 45-55°C and add directly to column. Let water sit for 5 minutes and centrifuge at 15,000 rpm for 30 seconds at RT.

1. Samples should be stored at -80°C until ready to be sent to Bioanalyzer for quality check.

**RNA Extraction Buffer Recipe:**

Reagents needed:

1% IGEPAL CA-630 (Solvay USA Inc. Lock Haven, PA)

100mM EDTA

0.02% SDS

20% b-mercapoethanol

0.5% Sodium azide

To make 100mLs:

1000 uL 1% IGEPAL (or Nonidet P40, IGEPAL substitute)

20 mL 0.5M EDTA

200 uL 10% SDS

20 mL b-mercapoethanol

0.5 g Sodium azide

Bring volume up to 100mL with DEPC water
