# Supplementary material for: The cacao gene atlas: a transcriptome developmental atlas reveals highly tissue-specific and dynamically-regulated gene networks in Theobroma cacao L
Source: BMC Plant Biol. 2024 Jun 26;24:601. doi: 10.1186/s12870-024-05171-9 (PMC11201900; doi:10.1186/s12870-024-05171-9)
Supplement: Supplementary file 10 — Additional File 10: Pearson Correlation of QuantSeq and TruSeq Counts [file 12870_2024_5171_MOESM10_ESM.docx]

**Additional File 10. Person Correlation of QuantSeq and TruSeq Counts.** Pearson correlation of DESeq2 regularized log transformed counts to present relative abundance transformation that accounts for both differing library sizes and stabilizes the variance among counts. The experimental design categorizes the QuantSeq and TruSeq as different groups so that variance stabilization is performed within groups because of differences in data volume.
